# Differentially expressed platelet activation-related genes in dogs with stage B2 myxomatous mitral valve disease

**DOI:** 10.1186/s12917-023-03789-9

**Published:** 2023-12-13

**Authors:** Qingqing Zhou, Xiang Cui, Han Zhou, Shuai Guo, Zhimin Wu, Liyang Li, Jinxin Zhang, Wen Feng, Yingfang Guo, Xiaofei Ma, Yu Chen, Changwei Qiu, Ming Xu, Ganzhen Deng

**Affiliations:** 1 Department of Clinical Animal Medicine, College of Animal Medicine, Huazhong Agricultural University, Wuhan, 430070 China; 2 Department of Clinical Animal Medicine, College of Veterinary Medicine, Gansu Agricultural University, Lanzhou, 730070 China

**Keywords:** Dog, Myxomatous mitral valve, Biomarkers, Transcriptomic, *MDM2*

## Abstract

**Background:**

Peripheral blood carries a reservoir of mRNAs that regulate cardiac structure and function potential. Although it is well recognized that the typical symptoms of Myxomatous Mitral Valve Disease (MMVD) stage B2 are long-standing hemodynamic disorder and cardiac structure remodeling caused by mitral regurgitation, the transcriptomic alterations in blood from such dogs are not understood.

**Results:**

In the present study, comparative high-throughput transcriptomic profiling of blood was performed from normal control (NC) and naturally-occurring MMVD stage B2 (MMVD) dogs. Using Weighted Gene Co-expression Network Analyses (WGCNA), Gene Ontology (GO), and Kyoto Encyclopedia of Gene and Genomes (KEGG), we identified that the turquoise module was the most highly correlated with echocardiographic features and found 64 differentially expressed genes (DEGs) that were significantly enriched in platelet activation related pathways. Therefore, from the turquoise module, we selected five DEGs *(MDM2*, *ROCK1*, *RIPK1*, *SNAP23*, and *ARHGAP35*) that, according to real-time qPCR, exhibited significant enrichment in platelet activation related pathways for validation. The results showed that the blood transcriptional abundance of *MDM2*, *ROCK1*, *RIPK1*, and *SNAP23* differed significantly (*P* < 0.01) between NC and MMVD dogs. On the other hand, Correlation Analysis revealed that *MDM2*, *ROCK1*, *RIPK1*, and *SNAP23* genes negatively regulated the heart structure parameters, and followed the same trend as observed in WGCNA.

**Conclusion:**

We screened four platelet activation related genes, *MDM2*, *ROCK1*, *RIPK1*, and *SNAP23,* which may be considered as the candidate biomarkers for the diagnosis of MMVD stage B2. These findings provided new insights into MMVD pathogenesis.

**Supplementary Information:**

The online version contains supplementary material available at 10.1186/s12917-023-03789-9.

## Background

Myxomatous mitral valve disease (MMVD) is the single most common acquired cardiac disease in dogs, and accounts for 75%–80% of cardiovascular disease in small breeds (≤ 20 kg) including Poodle, Cavalier King Charles Spaniel, and Chihuahua [1, 2]. Resultant mitral regurgitation (MR) can lead to chronic volume overload with left atrial dilatation, left ventricular eccentric hypertrophy, and mitral valve tissue remodeling even accompanied by prolapse [2, 3]. Initially, myocardial and mitral valve remodeling resulting from adaptive changes can enhance cardiac output to meet the metabolic demand of the organism, but congestive heart failure (CHF) is the most common sign by which MMVD progresses to end stage MMVD [4, 5]. Thus, clarification of the pathogenesis of hemodynamic disturbance is essential for identifying effective prevention strategies for MMVD.

Generally, a dynamic-static diagnosis mode, echocardiography in combination with radiography, is considered a more accurate way to detect cardiovascular disease [6, 7]. Moreover, the American College of Veterinary Internal Medicine (ACVIM) divided MMVD into four stages (stage A, B, C, and D) according to echocardiographic and radiographic results, including normalized LV internal diameter in diastole (LVIDDN), left atrial to aortic (LA:AO) ratio, and vertebral heart size (VHS). Stage B of MMVD identifies dogs with structural heart disease, but that have no clinical signs, also known as asymptomatic MMVD. Stage B of MMVD is divided into stage B1 and stage B2 according to whether there is radiographic and echocardiographic evidence of cardiac remodeling owing to MMVD. Stage B2 is the first period of cardiac remodeling, which can be detected by imaging, and it is also an important stage at which pharmacotherapy is required to delay the course of the disease [2, 8]. However, echocardiograph and radiography examinations are time-consuming and are subject to both method and operator-dependent errors [9]. Studies report that leptin, adiponectin, and natriuretic peptides (NT-ProBNP) in blood have been used as biomarkers of MMVD [1, 10]. However, dogs classified as MMVD stage C had serum concentrations of leptin and adiponectin higher than healthy dogs, but stage A or B dogs did not [1]. Furthermore, dogs of stages B1 and B2 could not be distinguished based on NT-ProBNP concentration [10]. Specific biomarkers for MMVD stage B2 have not been identified in peripheral blood, however, most omics studies on MMVD conducted to date have focused primarily on valvular tissue samples [11–13] and there is only a weak correlation between transcriptome levels in peripheral blood and tissue [14]. Additionally, conventional MMVD biomarkers exhibit low specificity and are susceptible to non-cardiac problems, which limits their usefulness in disease diagnosis, treatment, and prognostic evaluation [15]. In summary, there is an urgent need to identify novel biomarkers for MMVD stage B2.

Transcriptomics has developed in parallel with sequencing technology and bioinformation technology, and provides a useful method for revealing new biomarkers and their related functional properties [16, 17]. In addition, transcriptomic methods are more reproducible, more sensitive, and have a higher genome coverage than proteomic-based approaches [18]. Weighted Gene Co-expression Network Analyses (WGCNA) is the most common bioinformation method for finding key modules associated with clinical features [19]. In recent years, several studies have carried out biological analysis using WGCNA, providing new ideas for research on cardiovascular disease pathophysiology [20, 21].

In order to explore the biomarkers in MMVD stageB2 in the peripheral blood in dogs, in this prospective study, WGCNA was used to identify MMVD stage B2 clinical characteristic associated modules and hub genes. Furthermore, Gene Ontology (GO) and Kyoto Encyclopedia of Gene and Genomes (KEGG) analyses were conducted to assess potential functions of the differentially expressed genes (DEGs). The results of WGCNA combined with the results of function enrichment analysis of DEGs were used as a basis for selecting candidate biomarkers in a discovery cohort. The candidate biomarkers were then confirmed in a validation cohort using qPCR and correlation analysis. These results may help identify the clinical phenotype-related DEGs and mechanisms present during MMVD stage B2. Our study provides a novel strategy for researching the mechanisms of action of MMVD in dogs and developing genetic markers for MMVD stage B2.

## Results

### Animal characteristics

The characteristics of the animals used in this study are described in Additional file 1. While there was no difference in the sex and body weight of dogs in the NC and MMVD discovery cohorts (*P* > 0.05), age differed significantly between the NC and MMVD validation cohorts (*P* < 0.05). The radiology parameters, LA:AO, LVIDDN, and VHS, also differed significantly between NC and MMVD stage B2 dogs in both the discovery and validation cohorts.

### Quality control and RNA sequence data mapping

A total of 672.7 million raw data reads (approximately 201.6Gbps) were yielded across 8 samples. After filtering the raw data by removing those containing N (reads with more than 10% N), low-quality, and adaptor reads, 668.0 million clean reads (approximately 200.4Gbps, 23.1–27.7 Gbps for each sample) were retained and used for subsequent assembly and analysis. The mean values of Q20 and Q30 for clean reads across all samples were > 98.3% and > 95.2%, respectively. In comparison to the canine reference genome CanFam3.1, 95.8% and 95.5% of the total reads for the NC and MMVD groups, respectively, were mapped on the *Canis lupus familiaris* genome, with a GC content > 56.4%, indicating that the obtained clean data were high quality and the valid sequences could be used for subsequent analysis. The quality of the sequencing data for all samples in this experiment is summarized in Additional file 2.

### Analysis of gene expression level

In RNA-seq experiments, gene expression level is estimated by the abundance of transcripts that are mapped by the genome or exon, and FPKM was the most common method for evaluation. According to statistics, a total of 25,464 genes (including 884 novel genes) were identified, and approximately 84.6% of the genes with the FPKM values were below 3 in the NC and MMVD groups; few of the12.9% genes were highly expressed (the gene with FPKM value of 3–60), and only 2.5% genes were highly expressed with FPKM values > 60. The data are summarized in Additional file 3.

**Fig. 1:**
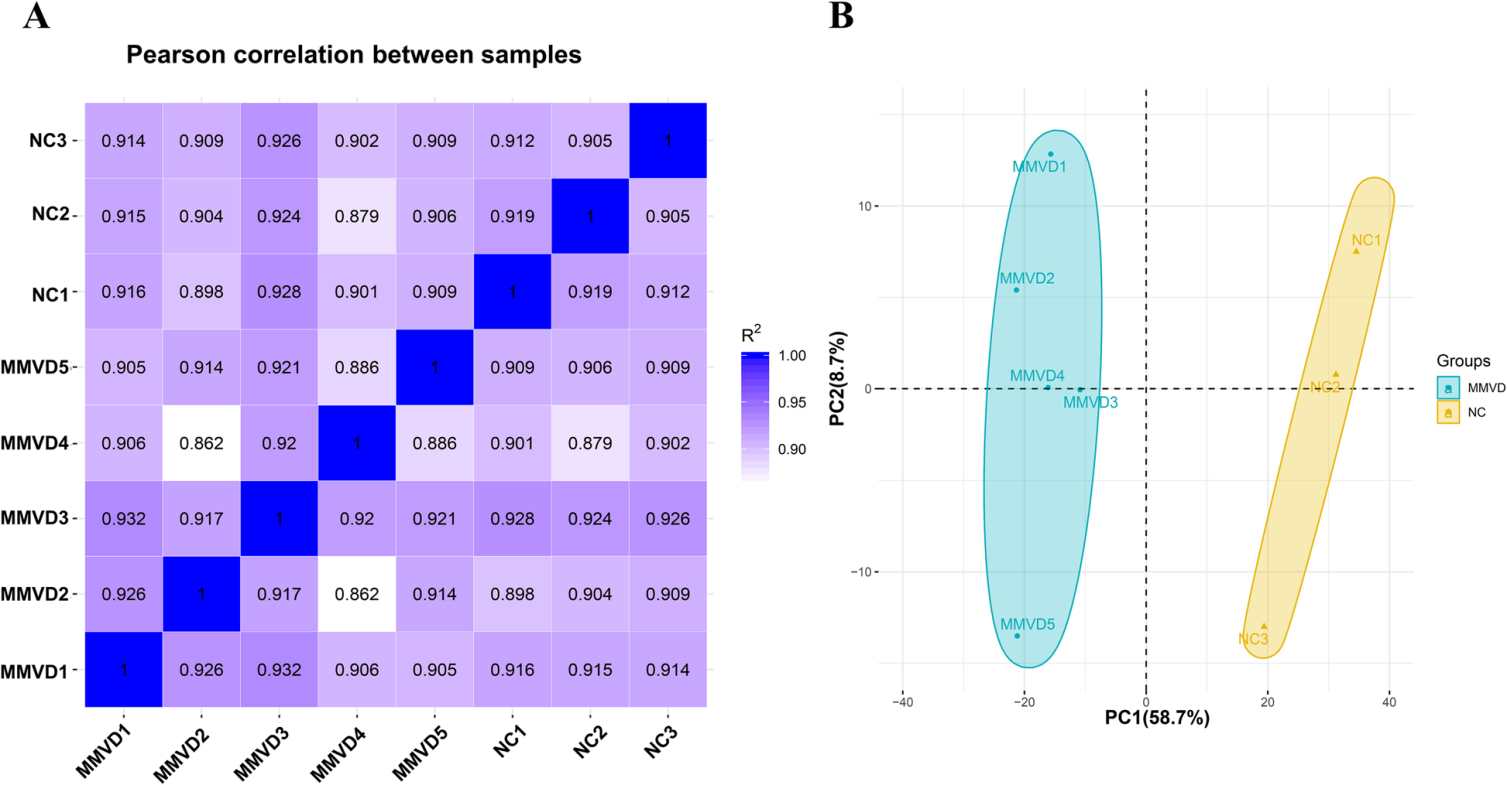
Correlation analysis and principal component analysis of the samples. **A** Heatmap of hierarchical clustering of eight samples (3 NC and 5 MMVD) using Pearson’s method. Bold values represent the R2 (square of the Pearson correlation coefficient) for replicates of each sample. All the P values are below 1e^−16^. **B** Principal component analysis plot of the samples. The NC group (yellow triangle) and MMVD group (green dot) samples are labelled with different shapes and colors, and a 95% confidence ellipse for each group is shown. The percentages represent the contribution ratio

According to all gene expression levels (FPKM) of all 8 samples, the correlation coefficient of samples between groups was calculated and drawn as a heat map (Fig. 1A). It can be intuitively shown that the correlation indices of the mapped genes in different groups were different, but the expression patterns within the groups were similar. To determine whether the samples were clustered based on MMVD, we performed PCA on the quantitative genes of the transcriptomics across all samples. The first and secondary principal components (PC1 and PC2) separated all samples, contributing 58.7% and 8.7% explanation of variance, respectively (Fig. 1B). Samples from each group were clustered together, indicating differences between the NC and MMVD groups, but with good sample repeatability within the groups. Gene expression level analysis indicated the reliability of the experiment and the plausibility of the sample selection, and provided a guarantee for subsequent differential gene analysis.

### Weighted correlation network analysis

**Fig. 2:**
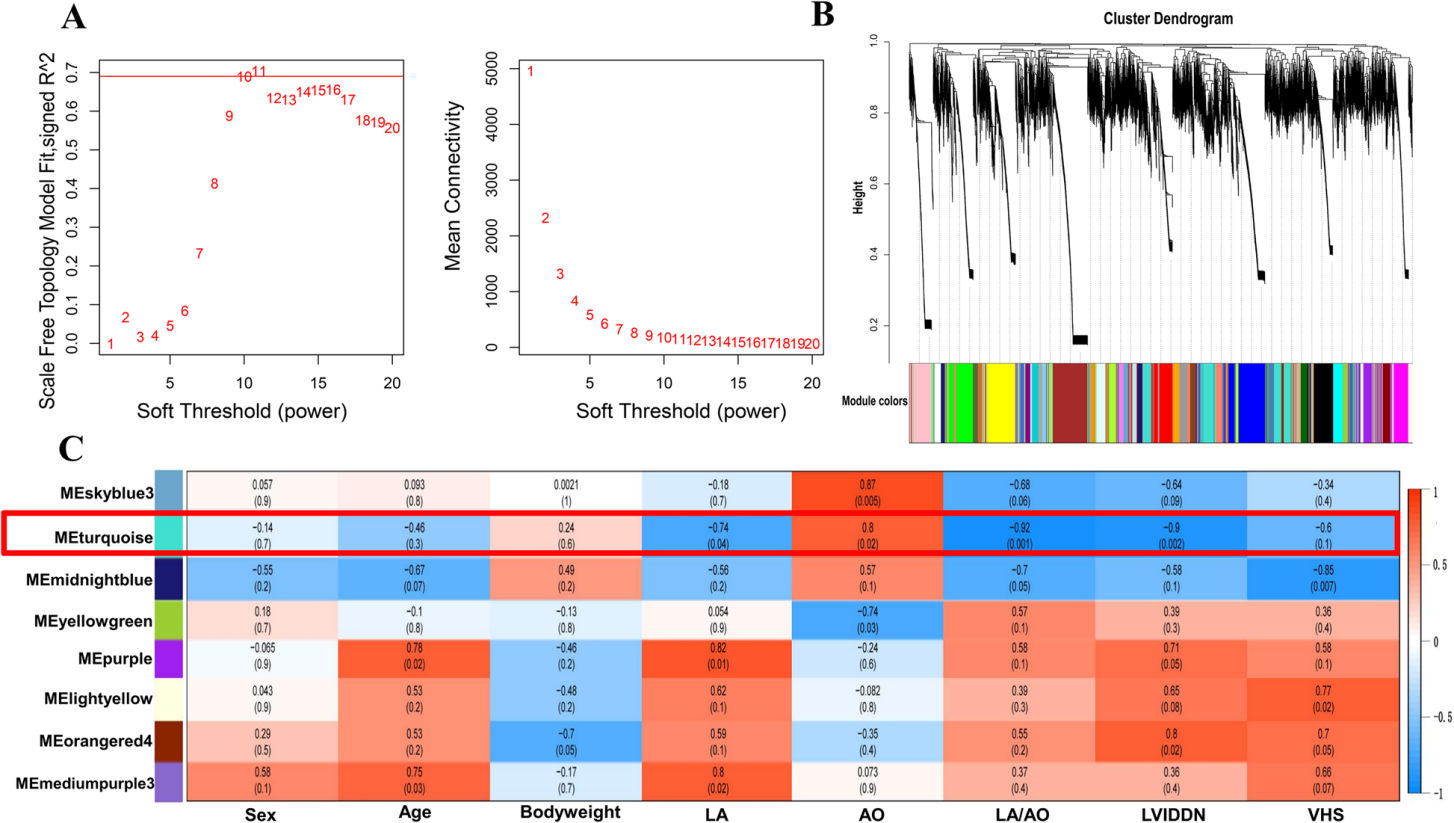
Key modules correlate with clinical traits through WGCNA. **A** Analysis of the scale-free fit index (left) and the mean connectively (right) for various soft-thresholding powers. **B** Clustering dendrogram of genes based on dissimilarity measures, together with assigned module colors. **C** Heatmap of the correlation between the module and clinical traits of MMVD. Each cell contains the correlation coefficient and *P*-value

Weighted correlation networks can be used to identify sets of genes that can be highly synergistically altered, as well as the correlations between gene sets and phenotypes. In order to find the key modules most relevant to the clinical characteristic of MMVD, we performed WGCNA on the transcriptomic database incorporating the identified genes. Clinical MMVD sample information included sex, age, and body weight, and radiology parameters, including LA, AO, LA/AO, LVIDDN, and VHS. By setting the soft-thresholding power as 10 (scale free R^2^ = 0.7), we eventually identified 41 modules; a gene cluster dendrogram is shown in Fig. 2A-B. From the heatmap of module trait correlations, eight modules were found to be strongly and significantly correlated with at least one parameter of clinical heart structure (Fig. 2C). Meanwhile, we identified the turquoise module as being highly positively correlated with AO (correlation coefficient was 0.80, *P* < 0.05); Meanwhile, turquoise module as being highly negatively correlated with LA, LA:AO, and LVIDDN (correlation coefficients were -0.74, -0.92, and -0.90, respectively, *P* < 0.05); moderately negative correlation with VHS (correlation coefficient was -0.60, *P* < 0.05); but lower correlations were shown with sex, age, and bodyweight (correlation coefficients were -0.14, -0.46, and 0.24 respectively, *P* > 0.0.5). and all modules are shown in Additional file 4**.** The turquoise module contained a total of 1324 genes. According to the gene-module k-Within coefficients, we selected the top 100 as “hub genes” from the turquoise module (see Additional file 5) for further study.

### Analysis of differentially expressed genes (DEGs)

**Fig. 3:**
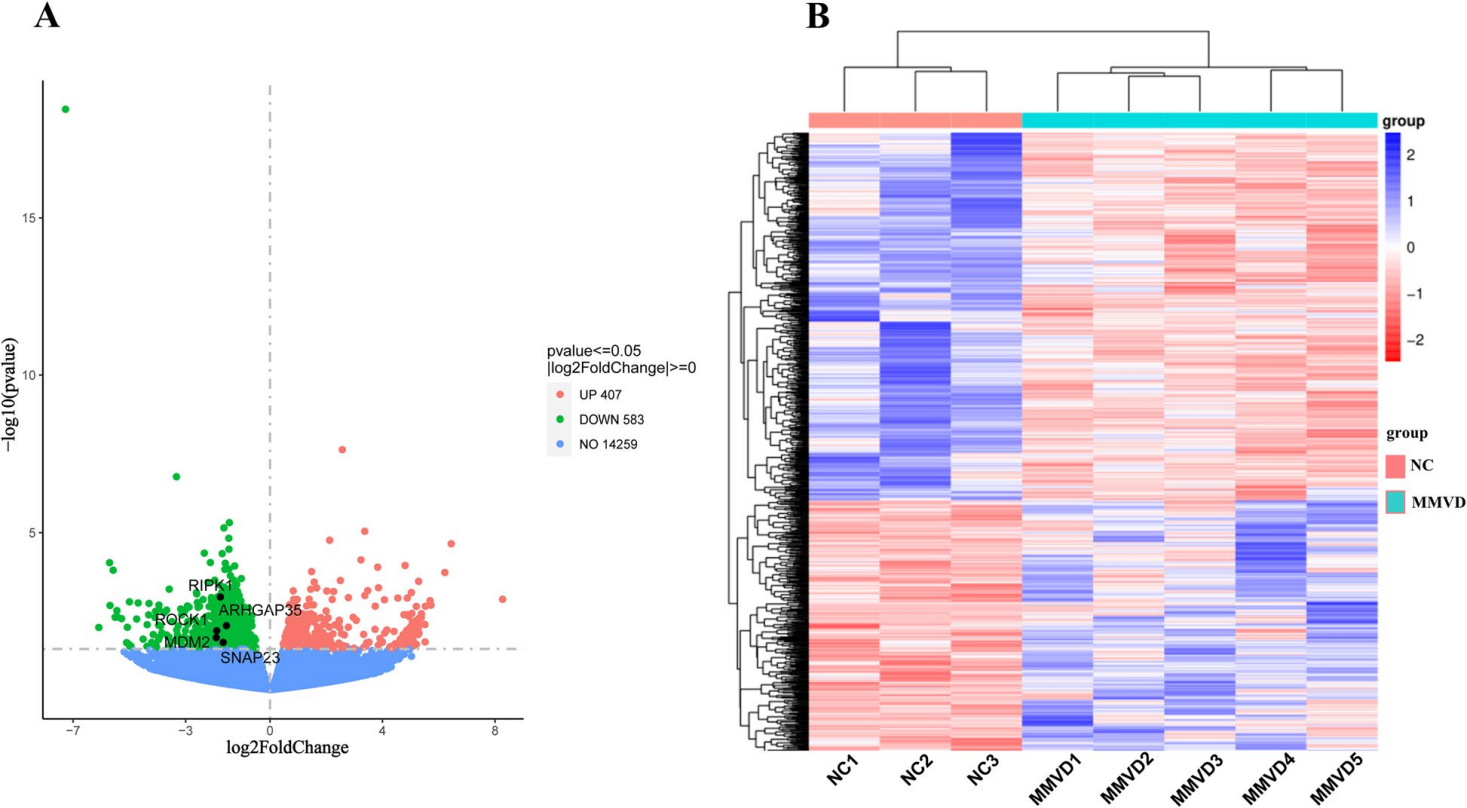
DEG identification and expression. **A** Volcano plot of the DEGs between in the NC and MMVD groups. The X-axis represents the fold change of DEG expression and the Y-axis represents the statistical significance of the fold change. Each dot represents DEG. Red dots represent DEGs with significant upregulation; green dots represent DEGs that are significantly downregulated, and blue dots represent DEGs that are not significant. **B** Cluster heatmap of the DEGs. The rows and columns represent genes and samples, respectively. The legend represents log2FC of gene abundance. Red and blue represent high and low expression, respectively

The read counts obtained from gene expression analysis are used for differential expression analysis. Based on the transcript∣log 2 (fold change)∣ > 0 and *P*-value (< 0.05), the DEGs were identified. A total of 990 DEGs (934 referenced genes and 56 novel genes) between the NC and MMVD groups were found, including 407 upregulated DEGs and 583 downregulated DEGs as plotted in the Volcano plot in Fig. 3A (see Additional file 6). The hierarchical cluster heatmap showed that expression profiles of DEGs were different between the NC and MMVD groups (Fig. 3B).

### Functional analysis of DEGs

**Fig. 4:**
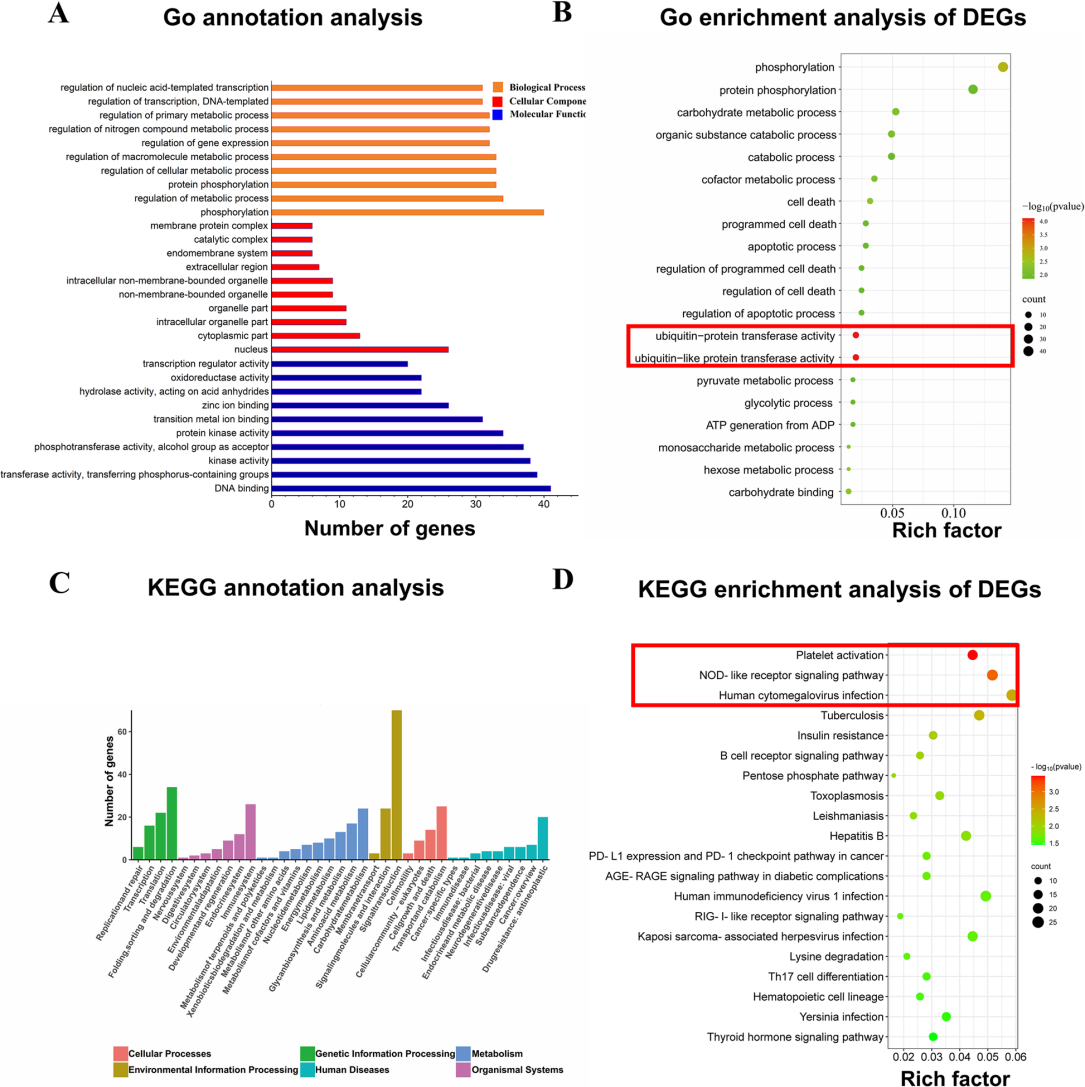
GO and KEGG enrichment analysis of the DEGs and Venn diagrams of candidate genes. **A** GO enrichment analysis of the DEGs. X-axis represents the top 10 GO terms and the Y-axis represents the statistical significance of fold change. BP (red) represents biological processes; CC (green) represents cellular components; and MF (blue) represents molecular functions. **B** The top 20 GO categories in the dogs with MMVD. **C** KEGG classification of 990 DEGs are summarized. **D **The top 20 KEGGs in dogs with MMVD

In order to better understand the function of DEGs, we performed GO analysis. The terms of phosphorylation (GO:0016310, 40DEGs), nucleus (GO:0005634, 26DEGs), and DNA-binding (GO:0003677, 41 DEGs) occupied the maximum proportion at the level of biological process (BP), cellular component (CC), and molecular function (MF), respectively (Fig. 4A). GO analysis results were considered statistically significant at *P*-value < 0.05. The top 20 GO terms for the DEGs are shown in Fig. 4B. The ubiquitin-protein transferase activity (GO: 0004882,* P*-value of 7.68E^−5^, 10 DEGs) and ubiquitin-like protein transferase activity (GO:0019787, *P*-value of 7.68E^−5^, 10 DEGs) terms classified into the MF class occupied the most significant enrichment degrees. The same 10 DEGs encoding ubiquitin protein ligase3 (*HERC3*), complex subunit 2 (*MSL2*), ubiquitin protein ligase E3 component n-recognin 5 (*UBR5*), thyroid hormone receptor interactor 12 (*TRIP12*), ubiquitin protein ligase E3A (*UBE3A*), and ubiquitin protein ligase E4A (*UBE4A*), and so on, were directly related to ubiquitin-protein and ubiquitin-like protein transferase activity.

Using KEGG functional annotations, the 990 DEGs were classified to identify the pathways in which they participated. Signal-transduction (70 DEGs), folding-sorting-degradation (34 DEGs), and immune system (26 DEGs) occupied the maximum proportion in environmental information processing, genetic information processing, and organismal systems, respectively (Fig. 4C). The most significant enrichment pathways are shown in Fig. 4D. The DEGs were mapped to 286 KEGG pathways, and the top 20 most common pathways were identified. The significantly regulated genes were mainly involved in the platelet activation pathway (KEGG: cfa04611, *P*-value of 3.47E^−4^, 19 DEGs), NOD-like receptor signaling pathway (KEGG: cfa04621, *P*-value of 7.68E^−4^, 22 DEGs), and human cytomegalovirus infection pathway (KEGG: cfa05163, *P*-value of 2.01E^−3^, 25 DEGs). According to the KEGG and GO analysis results, these 5 pathways included the ubiquitin-like protein transferase activity, ubiquitin protein transferase activity, platelet activation, NOD-like receptor signaling pathway, and human cytomegalovirus infection pathway. Among these pathways, a total of 64 DEGs were found, only 19 DEGs were upregulated, and the other 45 DEGs were downregulated (see Additional file 7).

**Fig. 5:**
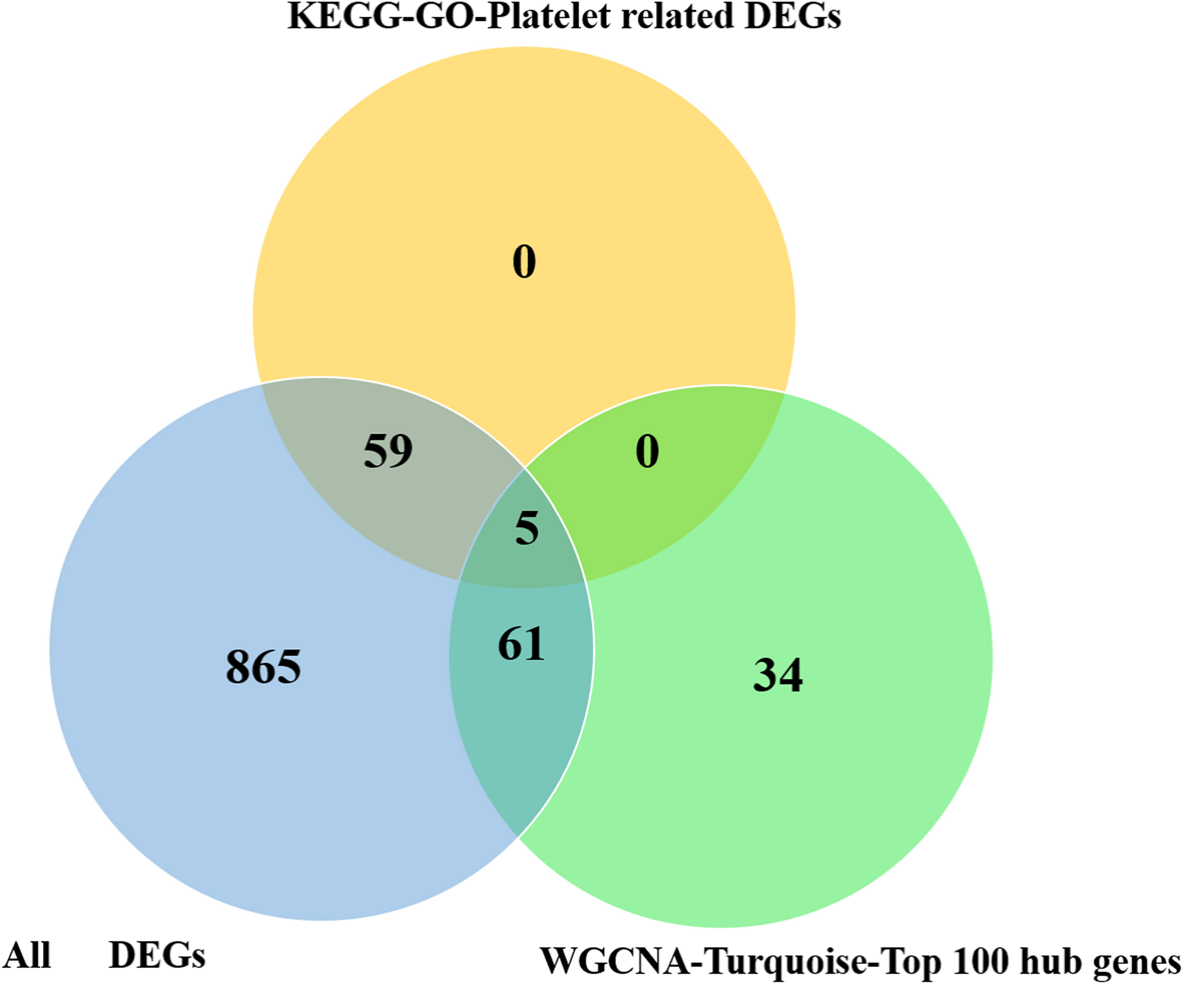
A Venn diagram of all DEGs, platelet-activation-related DEGs in KEGG and GO analysis and the top 100 hub genes in the turquoise module

In order to verify whether the hub genes within the turquoise module were related to the platelet activation-related DEGs, we drew a Venn diagram. Surprisingly, of these 100 “hub genes”, 66 genes belong to DEGs, and 5 genes belong to the platelet activation-related DEGs (Fig. 5), including *MDM2* proto-oncogene (*MDM2*, log2FC = -1.94, *P* < 0.05), Rho associated coiled-coli containing protein kinase 1 (*ROCK1*, log2FC = -1.92, *P* < 0.05), receptor interacting serine/threonine kinase 1 (*RIPK1*, log2FC = -1.79, *P* < 0.05), synaptosome associated protein 23 (*SNAP23*, log2FC = -1.69, *P* < 0.05), and Rho GTPase activating protein 35 (*ARHGAP35*, log2FC = -1.58, *P* < 0.05). Therefore, we selected the 5 genes as the candidate biomarkers for subsequent experimental validation.

### RT-qPCR validation of transcriptome data results

**Fig. 6:**
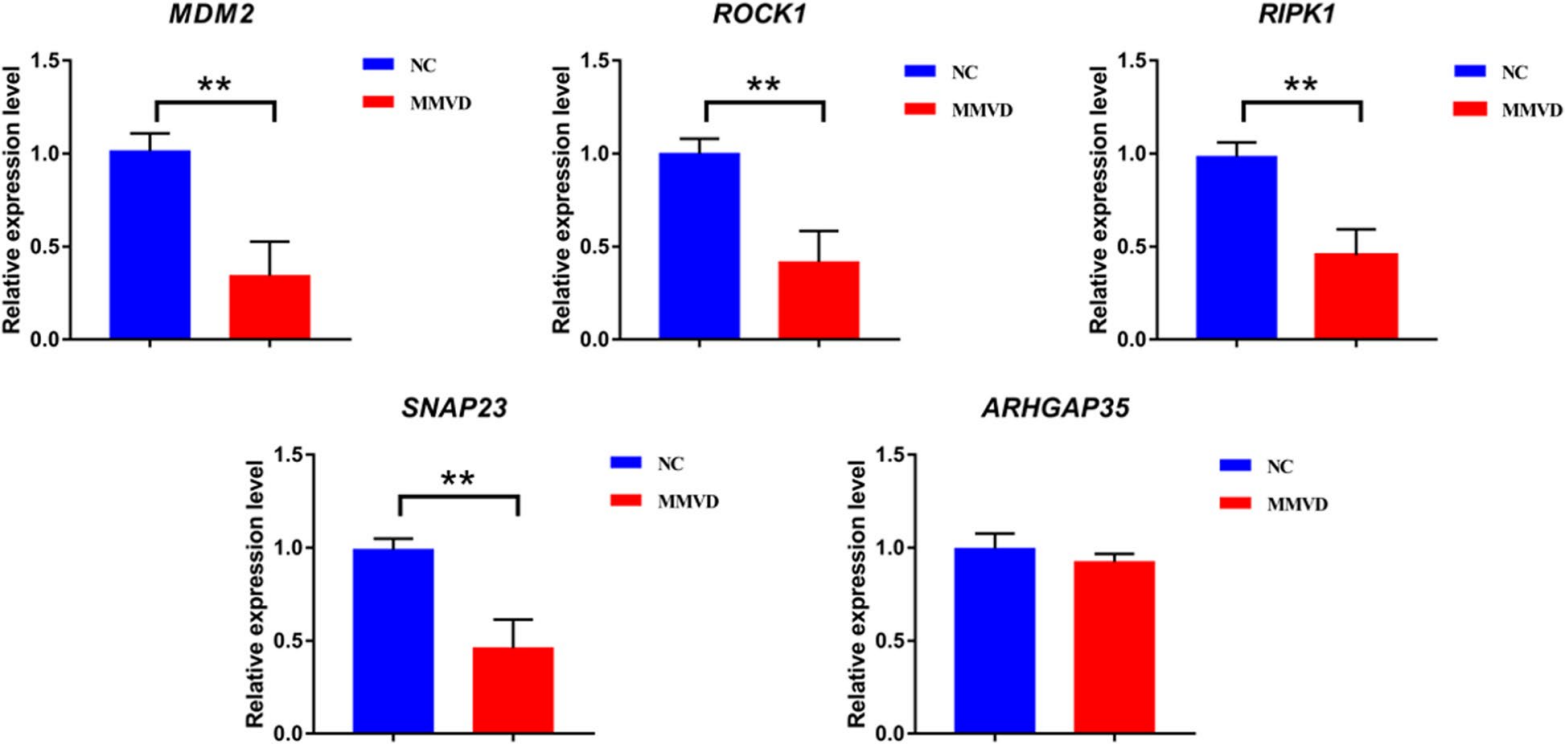
Real-time PCR validation of the candidate genes in the validation cohort. (**P < 0.01)

According to the differential expression analysis, functional enrichment analyses (KEGG and GO), and WGCNA results, 5 candidate DEGs (*MDM2*, *ROCK1*, *RIPK1*, *SNAP23*, and *ARHGAP35*) with the highest correlation with platelet activation related pathways and echocardiographic parameters were selected for validation in all 108 dogs (NC = 52; MMVD = 56) using RT-qPCR to validate the RNA-seq results. Here, statistically significant results were obtained for *MDM2*, *ROCK1*, *RIPK1*, and *SNAP23*. Compared with their expression in the NC group, *MDM2*, *ROCK1*, *RIPK1*, and *SNAP23* were down-regulated in the MMVD group and the result was significant (*P* < 0.01). *ARHGAP35* was obviously down-regulated, but the difference was not significant (*P* > 0.05). In summary, the expression patterns of the 5 candidate DEGs basically coincided with the RNA-seq (Fig. 6), indicating that *MDM2*, *ROCK1*, *RIPK1*, and *SNAP23* were potential MMVD biomarkers and drug targets.

### Correlation analysis of RT-qPCR results with radiology parameters of Dogs

**Fig. 7:**
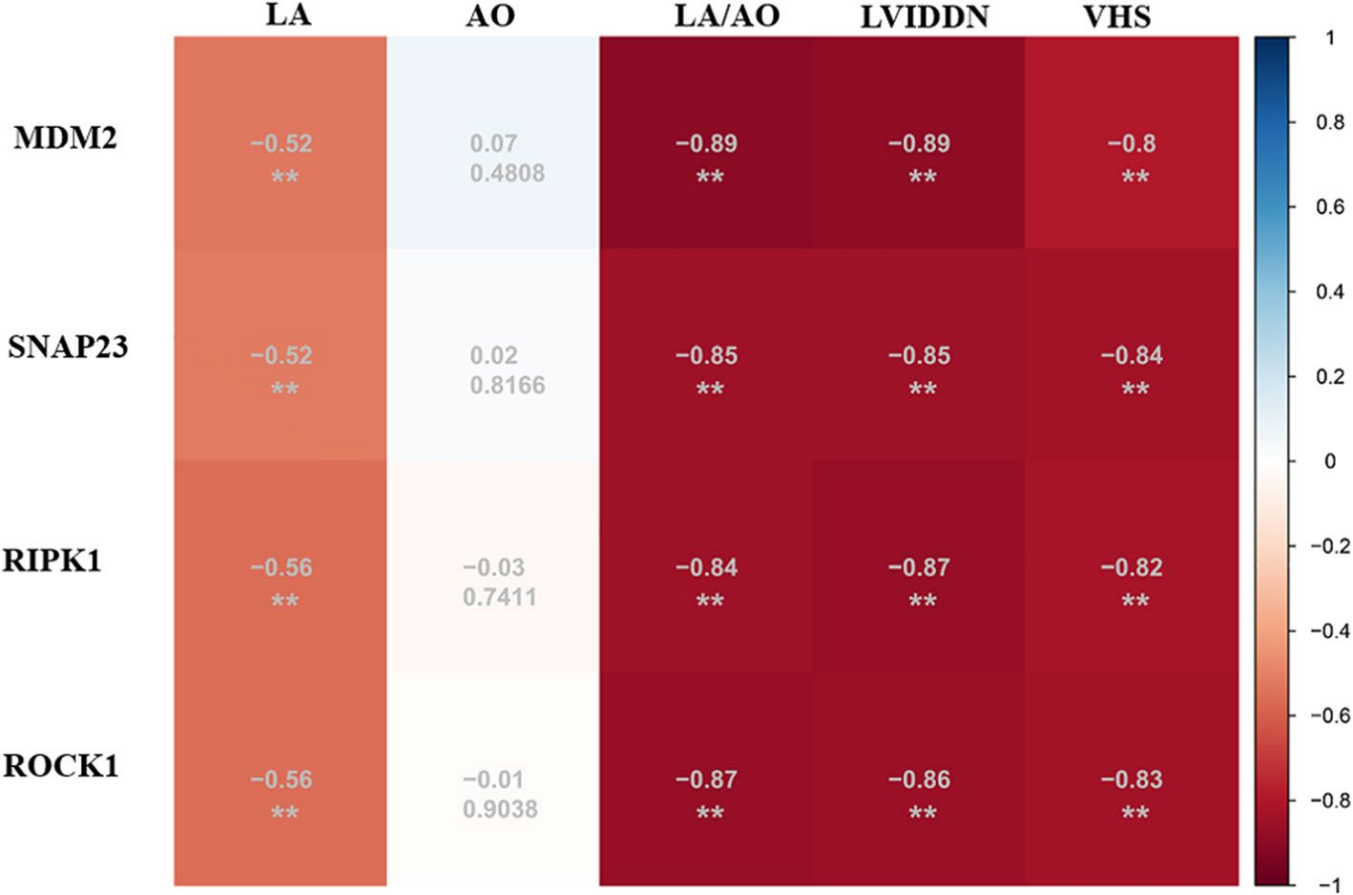
Correlation analysis between the radiology parameters and the candidate genes in the validation cohort (**P < 0.01)

To further determine the correlation of the 4 candidate genes with the radiology parameters, we correlated the RT-qPCR results of candidate genes in validation cohorts with radiology parameters. The results showed that *MDM2*, *ROCK1*, *RIPK1*, and *SNAP23* were significantly negatively correlated with LA/AO, LVIDDN, and VHS (*P* < 0.01). Correlation coefficients are shown in Fig. 7. It was found that LA/AO and LVIDDN had the highest correlation with *MDM2*, and the correlation coefficient was -0.89. There was a weak correlation between LA and the 4 candidate genes (the correlation coefficient was < -0.6). There was no correlation between AO and the 4 candidate genes. The results basically coincided with the WGCNA that were based on the results of RNA-seq, except that the LA and AO correlation coefficients were reduced.

## Discussion

Our study aimed to use RNA-seq combined with WGCNA systems biology method to identify blood biomarkers related to clinical features, that might inform the identification of diagnostic and therapeutic targets for MMVD stage B2. WGCNA is a systems biology method for identifying gene clusters, forming modules of highly correlated genes, and analyzing the relationships between modules and specific features [22]. WGCNA of MMVD has not yet been reported. The current study used WGCNA to identify biomarkers related to clinical traits, especially cardiac remodeling, that may help to identify novel diagnostic and therapeutic targets for MMVD stage B2.

A total of 25,464 genes identified from eight blood samples (three healthy control and five diagnosed with MMVD stage B2) were employed to construct 41 co-expression modules using WGCNA analysis. Through module-trait relationship analysis, the turquoise module was identified as being associated with clinical features (LA, AO, LA:AO, and LVIDDN) (Fig. 2C, Additional file 1), and LA:AO had the highest correlation coefficient. Interestingly, LA:AO is a convenient and body size-independent measurement, and it is also a typical index for evaluating the size of LA of dogs with MMVD [8, 23]. Thus, the turquoise module was used for subsequent analysis, because it is likely to correlate more closely correlated with cardiac remodeling. Through further analysis using gene-module k-Within coefficients, 100 hub genes that were closely related to cardiac remodeling were obtained and used for subsequent analyses.

Identification and functional analysis of DEGs are a common method for exploring biomarkers. Therefore, using the criteria of ∣log2(fold change)∣ > 0 and *P*-value < 0.05, a total of 990 DEGs were identified, including 583 upregulated DEGs and 407 downregulated DEGs. To study the mechanism of the DEGs, functional analysis was performed. KEGG and GO function analyses (Fig. 4B, D) found that DEGs were significantly enriched in platelet activation, NOD-like signaling, human cytomegalovirus infection, and ubiquitin and ubiquitin-like protein transferase activity. Compared to those in the peripheral blood transcriptome, most DEGs in the valvular tissue transcriptome of dogs with MMMVD were significantly enriched in epithelial-to-mesenchymal transition, basement membrane components, and transforming growth factor-β signaling [11–13, 24]. Inflammation, extracellular matrix organization and platelet function-related pathways were significantly enriched in both the peripheral blood and tissue transcriptomes. These findings suggest that genes associated with inflammation, extracellular matrix organization, and platelet activation may play a role in MMVD pathogenesis.

Platelet activation is associated with NOD-like receptors, Human cytomegalovirus infection and ubiquitin-like protein transferase activity [25–27]. However, the platelet-related pathways have extensive attention owing to its involvement in a variety of cardiac disease processes [28, 29]. NOD-like receptor signaling is shown to induce platelet activation during heatstroke [30] and sepsis [25]. Human cytomegalovirus directly activates the platelet-derived growth factor system, and human cytomegalovirus-platelet interactions lead to proinflammatory and proangiogenic responses, that exacerbate tissue damage and contribute to atherogenesis [31, 32]. Previous studies have shown extensive protein ubiquitylation during of platelet activation [33–35]. These results demonstrated that platelet activation correlates with NOD-like signaling, human cytomegalovirus infection, and ubiquitin and ubiquitin-like protein transferase activity pathways, indicating that platelet activation-related genes play a major role in the development of MMVD. Thus, the DEGs significantly enriched in these pathways were defined as platelet activation-related DEGs for subsequent analysis.

Platelets are discoid, anucleate cells released into the bloodstream from megakaryocytes [36]. They play an important role in the development of cardiovascular diseases such as atherosclerosis, myocardial infarction, and diabetic coronary heart disease [37–39]. Studies on MMVD progression in dogs have shown that turbulent high-velocity blood flow and changes in blood shear stress around the mitral valve leaflets may produce platelet activation [24, 28]. Moreover, in a recent study of changes in the proteome of platelets in dogs with MMVD-related acute congestive heart failure, most (60%) of the differentially expressed proteins were down-regulated [28]. The current study similarly found that 45 of 64 (70%) platelet activation-related DEGs were down-regulated. It is intriguing that several platelets activation-related genes were significantly lower in dogs with MMVD. Further research using larger studies is needed to identify potential mechanisms for this effect.

It is worth noting that of the top 100 hub genes in the turquoise module, five hub genes (*MDM2*, *ROCK1*, *RIPK1*, *SNAP23*, and *ARHGAP35*) jointly participated in five platelet-related biological processes or pathways. The RNA-seq and bioinformatics results indicated that biological processes and pathways related to platelet-related have an important impact on the diagnosis of MMVD stage B2.

The *MDM2* proto-oncogene (*MDM2*) is a RING domain-containing E3 ubiquitin ligase [40]. Previous studies have shown that *MDM2* is involved in cancer cell proliferation by activating platelet-derived growth factor and deubiquitinating, and is overexpressed in many soft-tissue sarcomas, such as osteosarcoma [41], retroperitoneal liposarcoma [42], and liposarcoma [43]. A decreased platelet count is the most common abnormality, when patients with local or metastatic solid tumors are treated with an antagonist of *MDM2 *[44–46]. Moreover, circulating low platelet counts in certain MMVD-susceptible dog breeds, such as the Cavalier King Charles Spaniel and Maltase dogs, are thought to represent a familial feature that could result in the early onset of MMVD [47, 48]. In the current study, *MDM2* expression was significantly lower in dogs with MMVD stage B2 than in healthy dogs (Fig. 5). In addition, the *MDM2* mRNA expression was significantly negatively correlated with LA:AO, LVIDDN, and VHS (correlation coefficient = -0.89, -0.89 and -0.80, respectively, *P* < 0.01) (Fig. 6). Overall, these findings suggested that *MDM2* may be a promising diagnostic and therapeutic target for MMVD stage B2. Unfortunately, an analysis of platelet count and function was not performed in the current study. Thus, the potential relationship between the significant down-regulation of *MDM2* in MMVD and the number and activation status of platelets requires further investigation.

*ROCK1*, also known as Rho-kinase B, is a serine kinase as well as an important effector of RhoA GTPases [49]. Importantly, RhoA GTPases are master orchestrators of platelet physiology by inducing the vascular effects of various vasoactive factors to promote hemostasis, and play a major role in the development of cardiovascular disease [50, 51]. *ROCK1/2* inhibition increased megakaryocytes ploidy, aberrant proplatelet release, and macrothrombocytopenia [52–54]. Interestingly, another study found that both macrothrombocytopenia and an increase in LV fractional shortening were associated with a more hypercoagulable hemostatic system in dogs with MMVD. This study also showed a reduction in platelet activation in dogs with macrothrombocytopenia [55]. In the current study, *ROCK1* was significantly down-regulated in dogs with MMVD stage B2 compared to healthy dogs (Fig. 5). Thus, ROCK1 may contribute to the development of MMVD stage B2 by inhibiting platelet activation. In addition, the current study also identified two new targets, including *RIPK1* and *SNAP23*, that have not been previously been studied in MMVD. These results, using qPCR and correlation analysis with cardiac remodeling parameters, confirmed that they were differentially expressed in MMVD stage B2 and were significantly associated with cardiac remodeling induced by MMVD stage B2.

The current study had some limitations. First, a larger sample size is needed to obtain more reliable results, especially in the discovery cohort. This may explain why *ARHGAP35* was differentially expressed in the RNA-seq analysis, but not in the multi-breeds validation trials. Second, using only one breed (Poodle) in the discovery cohort is a major limitation. In addition, the current study was a preliminary exploration of genes involved in MMVD stage B2. A more in-depth comparison of dogs with MMVD stages B1 and B2 and the healthy dogs is still needed. A potential limitation of this study must be acknowledged, as no platelet count and/or its functions were studied. Further exploration of the molecular mechanisms by which these platelet activation-related hub genes impact MMVD development both in vivo and in vitro is also required.

## Conclusions

In conclusion, we employed RNA-sequencing technology combined with bioinformation analysis methods to identify gene co-expression modules associated with MMVD stage B2 cardiac remodeling and to reveal the potential hub genes and molecular mechanisms related to platelet activation in the case of MMVD stage B2. As far as we know, this is the first study to use WGCNA analysis in studying the clinical features of dogs with MMVD stage B2. Additionally, we identified 4 new MMVD stage B2 targets *(MDM2*, *ROCK1*, *RIPK1*, and *SNAP23*) that have been studied in many cardiovascular diseases in human medicine, such as chronic heart failure, atherosclerosis, and diabetic cardiomyopathy. However, they have not previously been studied in MMVD, and we validated their differential expression in MMVD stage B2. The results provide a rationale for deeper investigations into the involvement of *MDM2*, *ROCK1*, *RIPK1*, and *SNAP23* in the pathogenesis of MMVD.

## Methods

### Animals and blood collection

All the dogs were patients during August 2020 and December 2021 at the Huazhong Agricultural University’s Veterinary Medical Teaching Hospital (Wuhan, China). No dogs were euthanized for the purpose of the study and all blood was collected and used with full informed written owner consent, and the Animal Care and Use Committee of the Huazhong Agricultural University (HZAUMO-2015–12) approved all dog-related procedures in this study. After blood and clinical data collection, the dogs were returned to their owners.

According to the ACVIM consensus guideline for the diagnosis and treatment of myxomatous mitral valve disease in dogs, 118 enrolled dogs were divided into either the normal control group (NC) or naturally-occurring MMVD stage B2 group (MMVD), of which two did not meet our inclusion and exclusion criteria because of extreme aggressiveness during blood collection [8]. Finally, a discovery cohort (three NC and five with MMVD stage B2 Poodles) and a validation cohort (52 NC and 56 with MMVD stageB2 multi-breed canines) were included in the study. Dogs that showed no abnormalities at the physical, clinical, and analytical examinations (mainly consisting of digital radiography and transthoracic echocardiography analyses) were categorized as normal controls. Inclusion criteria for MMVD stage B2 dogs were as follows: (1) murmur intensity ≥ 3/6; (2) echocardiography LA:AO ratio in the right-sided short axis view in early diastole ≥ 1.6; (3) radiographic vertebral heart score (VHS) > 10.5; (4) One of the 4 criteria that identify advanced Stage B2 in dogs is an increase in their left ventricular chamber size, such that normalized to their body weight (BW), it is ≥ 1.7; all these criteria needed to be met before inclusion in the study. Exclusion criteria were: (1) aggressive behavior; (2) serious disease of the liver, lung, kidney, and other important organs; (3) complication due to malignant tumor; and (4) dog have received any medicines within 30 days. For the calculations, the averaged measurements of echocardiographic parameters of three cardiac cycles were used. Imaging procedures were independently and separately assessed by two board-certified veterinary radiologists from the Huazhong Agricultural University’s Veterinary Medical Teaching Hospital who were blinded to the experiment.

Whole blood was collected from the jugular vein in tubes containing EDTA, and immediately mixed with TRIzol reagent (Ambion, Thermo Fisher Scientific, USA) at a ratio of 1:3, until the floccules disappeared, incubated at room temperate for 10 min, and stored at -80℃ for further analysis. All samples were analyzed within 12 months after collection.

### RNA isolation and library construction

Total RNA was extracted using TRIzol reagent according to the manufacturer’s instructions. Then, total amounts and integrity of RNA were assessed using an RNA Nano 6000 Assay Kit of the Bioanalyzer 2100 system (Agilent Technologies, CA, USA) [56]. Briefly, mRNA was purified from the total RNA using poly-T oligo-attached to magnetic beads. Fragmentation was carried out using divalent cations under elevated temperature in First Strand Synthesis Reaction Buffer (5X). The first strand of cDNA was synthesized using a random hexamer primer and M-MuLV Reverse Transcriptase, followed by RNA degradation using RNaseH. Second strand cDNA synthesis was subsequently performed using DNA Polymerase I and dNTP. Remaining overhangs were converted into blunt ends via exonuclease/polymerase activities. After adenylation of 3' ends of DNA fragments, adaptors with hairpin loop structure were ligated to prepare for hybridization. In order to select cDNA fragments of preferentially 370 ~ 420 bp in length, the library fragments were purified using the AMPure XP system (Beckman Coulter, Beverly, USA). After PCR amplification, the PCR product was purified with AMPure XP beads, and the resulting library was finally obtained. The library was quantized by a Qubit2.0 Fluorometer, then diluted to 1.5 ng/ul, and the insert size of the library was detected by an Agilent 2100 Bioanalyzer. The libraries were individually run-on single lanes for 150 cycles (paired-end) on a NovaSeq 6000 (Illumine Inc) [57].

### Genome alignment and gene annotation

Raw reads were analyzed by in-house perl scripts for quality control, and high-quality reads were obtained by removing reads containing adapters, reads containing Nbase, and low-quality reads. At the same time, Q20, Q30, and GC content of the clean data were calculated [58]. An index of the reference genome (http://asia.ensembl.org/Canis_lupus_familiaris/Info/Index) was built using Hisat2 (v.2.0.5) and paired-end clean reads were aligned to the reference genome using Hisat2 (v.2.0.5). The mapped reads of each sample were assembled by String Tie v1.3.3b (http://ccb.jhu.edu/software/stringtie/) in a reference-based approach for novel transcript prediction. FeatureCounts v.1.5.0-p3 was used to count the read numbers mapped to each gene. The genes were annotated by Basic Local Alignment Search Tool (BLAST) based on GO and KEGG databases.

### Analysis of gene expression levels and identification of differentially expressed genes

Feature Count v.1.5.0-p3 was used to count the reads number mapped to each gene. The Reads Per Kilobase Million (FPKM) of each gene was then calculated based on the length of the gene and the number of reads counts mapped to that gene [59]. Differential expression analysis of the two groups was performed using the DESeq2R package (1.20.0) based on raw feature counts of each gene. DESeq2 provides statistical routines for determining differential expressions in numerical gene expression data using a model based on a negative binomial distribution. The resulting *P*-values were adjusted using the Beniamini and Hochberg’s approach for controlling the false discovery rate, *P*-adj ≤ 0.05 and |log2(foldchange)|≥ 0 were set as the thresholds for significant differential expression and the DEGs were identified [60].

### Differential gene clustering analysis

We first performed a logarithmic transformation on the gene expression values (FPKM expression matrix) of all samples to approximate the data to an ideal normal distribution. Next, the Euclidean distance analysis method was applied to calculate the distances between all transformed data points. Finally, using the complete clustering method within hierarchical clustering, N objects were classified into k clusters, where objects within each cluster are mutually similar. The hierarchical clustering algorithm mainly consists of three steps, as follows: Initially, each point is considered as a cluster by itself. Next, the two clusters (or points, at the beginning) with the shortest distance between them are identified, and they are merged into a single cluster. The distance between two clusters refers to the distance between the closest points in each cluster. Finally, repeat the second step until all points are clustered into a single group.

### GO and KEGG enrichment analysis of DEGs

GO enrichment and KEGG analysis of differentially expressed genes were implemented using the cluster Profiler R package (3.8.1) (http://www.geneontology.org; http://www.genome.jp/kegg/, respectively). GO terms and KEGG pathways with corrected *P*-values < 0.05 were considered to be significantly enriched by DEGs. The top GO categories and KEGG pathways were selected based on their *P*-values.

### Weighted correlation network analysis

We extracted all 25,464 identified genes to perform WGCNA with the expression data retrieved from the transcriptome sequencing, which could be used as gene expression data by FPKM. In order to select candidate biomarkers, R package WGCNA was performed to find clinical trait-related modules and hub genes. We set soft-thresholding power as 10 (scale free R^2^ = 0.70), and minimal module sizes as 10 to identified modules. Key modules with the highest correlation (according to the Pearson correlation coefficient and *P*-value) with clinical traits was selected for further analysis. Hub genes are indicated by their high K_ME_ (eigengene connectivity) value.

### Validation of selected transcripts by RT-qPCR

Total RNA was extracted using TRIzol reagent (Invitrogen, USA), following the manufacturer’s instructions, and then reverse transcribed into cDNA using the HiScript IIReverse Transcriptase (Vazyme, Nanjing, China). Then, Real-time quantitative (RT-q) PCR was performed using the SYBR green Master Mix run on a Stepone Plus Real-Time PCR System (Roche LightCycler@96, USA). The relative expression levels of mRNAs were normalized to glyceraldehyde 3-phosphate dehydrogenase (GAPDH) following the 2-ΔΔCt comparative method. The primer sequences were designed using Primers Premiers 5.0 and the primers are listed in Additional file 8.

### Statistical analysis

Statistical analysis was performed using GraphPad Prism (v.8.0.2.236). Data were presented as means ± SEM. Comparisons of continuous variables between groups used the Student’s t-test as indicated. Differences were considered statistically significant when a two-side *P*-value was < 0.05. All experiments were performed 3 times.

### Supplementary Information

**Additional file 1.** Demographic and clinical data for the dogs included in the study.

**Additional file 2.** Sequencing data quality summary.

**Additional file 3.** Statistics of comparing with reference genome.

**Additional file 4.** Module-trait relationships.

**Additional file 5.** The Gene information of the top 100 “hub genes” in turquoise module.

**Additional file 6.** The list of top 50 upregulated and downregulated DEGs.

**Additional file 7.** The Gene information of platelet-related in KEGG and GO analysis.

**Additional file 8.** Primers used for qPCR.

## Data Availability

The raw sequence data reported in this paper have been deposited in the Genome Sequence Archive in BIG Data Center (Members and Partners, 2022), Beijing Institute of Genomics (BIG), Chinese Academy of Sciences, under accession numbers CRA008836, which can be publicly accessible at.

## Abbreviations

MMVD: Myxomatous mitral valve disease
cTNI: Cardiac troponin-I
DEGs: Differentially expressed genes
WGCNA: Weighted gene co-expression network analyses
LA:AO: Ratio of left atrium to aortic internal diameter in right-sided short axis view in early diastole
LVIDDN: Left ventricular internal diameter in diastole, normalized for body weight
NT-ProBNP: Natriuretic peptides
VHS: Radiographic vertebral heart score
MDM2: MDM2 proto-oncogene
ROCK1: Rho associated coiled-coli containing protein kinase 1
RIPK1: Receptor interacting serine/threonine kinase 1
*SNAP23*: Synaptosome associated protein 23
ARHGAP35: Rho GTPase activating protein 35
