# Supplementary material for: Differentially expressed platelet activation-related genes in dogs with stage B2 myxomatous mitral valve disease
Source: BMC Vet Res. 2023 Dec 13;19:271. doi: 10.1186/s12917-023-03789-9 (PMC10717932; doi:10.1186/s12917-023-03789-9)
Supplement: Supplementary file 1 — Additional file 1. Demographic and clinical data for the dogs included in the study. [file 12917_2023_3789_MOESM1_ESM.docx]

**Additional file 1:** Demographic and clinical data for the dogs included in the study

| Variable | | Discovery Cohort | | | Validation Cohort | | |
| --- | --- | --- | --- | --- | --- | --- | --- |
|  | NC(n=3) | | MMVD (n=5) | *P* | NC(n=52) | MMVD(n=56) | *P*-value |
| Sex(M/F) | 2/1 | | 2/3 | _- | 25/27 | 27/29 | _- |
| Age(years) | 3.1±1.2 | | 7.6±1.3 | 0.052 | 5.3±2.3 | 8.7±2.7 | ＜0.0001 |
| BW weight (Kg) | 6.0±1.1 | | 4.4±0.6 | 0.177 | 7.8±3.7 | 7.6±2.6 | 0.906 |
| Murmur intensity | _- | | Ⅲ~Ⅳ | _- | _-~Ⅰ | Ⅲ~Ⅳ | _-  ASDYG VHGDFHGFhjuf |
| LA | 1.6±0.1 | | 1.9±0.2 | 0.054 | 1.7±0.4 | 2.2±0.3 | ＜0.0001 |
| AO | 1.3±0.1 | | 1.0±0.1 | 0.038 | 1.2±0.2 | 1.2±0.2 | 0.967 |
| LA/AO | 1.3±0.2 | | 1.8±0.1 | 0.003 | 1.4±0.1 | 1.8±0.1 | ＜0.0001 |
| LVIDDN | 1.4±0.1 | | 1.9±0.1 | 0.005 | 1.4±0.1 | 1.8±0.1 | ＜0.0001 |
| VHS | 9.8±0.3 | | 11.0±0.3 | 0.003 | 9.9±0.3 | 11.0±0.3 | ＜0.0001 |

Note: The left atrium-to-aorta ratio (LA/AO), obtained from the right parasternal short-axis view; the left ventricular end-diameter normalized for the body weight (LVIDDN), measured on the M-mode obtained from the right parasternal short-axis view[1]. The VHS was estimated by placing both lines of the short and long axis over the thoracic spine starting at the fourth thoracic vertebra (T4) and counting the vertebral bodies as units to the nearest 0.1v. The short axis was placed perpendicular to the long axis so that its caudal edge terminated at the intersection point of the caudal cardiac silhouette with the ventral border of the vena cava[2].

1. Vezzosi T, Grosso G, Tognetti R, Meucci V, Patata V, Marchesotti F, Domenech O: **The Mitral INsufficiency Echocardiographic score: A severity classification of myxomatous mitral valve disease in dogs**. *J Vet Intern Med* 2021, **35**(3):1238-1244.

2. Wiegel PS, Mach R, Nolte I, Freise F, Levicar C, Merhof K, Bach JP: **Breed-specific values for vertebral heart score (VHS), vertebral left atrial size (VLAS), and radiographic left atrial dimension (RLAD) in pugs without cardiac disease, and their relationship to Brachycephalic Obstructive Airway Syndrome (BOAS)**. *PLoS One* 2022, **17**(9):e0274085.
