# Supplementary material for: Differentially expressed platelet activation-related genes in dogs with stage B2 myxomatous mitral valve disease
Source: BMC Vet Res. 2023 Dec 13;19:271. doi: 10.1186/s12917-023-03789-9 (PMC10717932; doi:10.1186/s12917-023-03789-9)
Supplement: Supplementary file 2 — Additional file 2. Sequencing data quality summary. [file 12917_2023_3789_MOESM2_ESM.docx]

**Additional file 2:** Sequencing data quality summary

| Sample | Clean reads(M) | Clean bases (Gb) | Error rate (%) | Q20(%) | Q30(%) | GC content (%) | Total mapping rate (%) |
| --- | --- | --- | --- | --- | --- | --- | --- |
| MMVD 1 | 164.6 | 24.7 | 0.02 | 98.59 | 95.81 | 57.01 | 96.9 |
| MMVD 2 | 158.7 | 23.8 | 0.02 | 98.51 | 95.66 | 56.86 | 97.6 |
| MMVD 3 | 184.8 | 27.7 | 0.02 | 98.45 | 95.47 | 57.14 | 97.3 |
| MMVD 4 | 181.5 | 27.2 | 0.02 | 98.33 | 95.24 | 57.53 | 95.5 |
| MMVD 5 | 164.6 | 24.7 | 0.02 | 98.61 | 95.88 | 56.95 | 97.5 |
| NC1 | 162.4 | 24.4 | 0.02 | 98.46 | 95.52 | 57.01 | 96.5 |
| NC2 | 165.3 | 24.8 | 0.02 | 98.49 | 95.6 | 56.44 | 97.3 |
| NC3 | 154.2 | 23.1 | 0.02 | 98.38 | 95.33 | 57.47 | 95.8 |

NOTE: Clean reads, clean data are reads count filtered from raw data. Statistics method is similar with raw reads. All the following analysis is based on clean data; Clean bases: Base number of raw data after filtering (number of clean reads) * (sequence length), converting unit to G; Error rate (%): base error rate of whole sequencing; Q20(%): The percentage of the bases whose Q Phred values is greater than 20. (Number of bases with Q Phred value > 20) / (Number of total bases) *100; Q30(%): The percentage of the bases whose Q Phred values is greater than 30. (Number of bases with Q Phred value > 30) / (Number of total bases) *100; GC content (%): The percentage of G&C base numbers of total bases (G&C base number) / (Total base number) *100.
