# Supplementary material for: Differentially expressed platelet activation-related genes in dogs with stage B2 myxomatous mitral valve disease
Source: BMC Vet Res. 2023 Dec 13;19:271. doi: 10.1186/s12917-023-03789-9 (PMC10717932; doi:10.1186/s12917-023-03789-9)
Supplement: Supplementary file 3 — Additional file 3. Statistics of comparing with reference genome. [file 12917_2023_3789_MOESM3_ESM.docx]

**Additional file 3:** Statistics of comparing with reference genome

| FPKM Internal | MMVD 1 | MMVD 2 | MMVD 3 | MMVD 4 | MMVD 5 | NC1 | NC2 | NC3 |
| --- | --- | --- | --- | --- | --- | --- | --- | --- |
| 0-1 | 59416 | 59442 | 59528 | 59298 | 59482 | 59035 | 59221 | 59238 |
| 1-3 | 2161 | 2075 | 2239 | 2236 | 2030 | 2470 | 2325 | 2295 |
| 3-15 | 2277 | 2268 | 2419 | 2403 | 2264 | 2549 | 2385 | 2401 |
| 15-60 | 1040 | 1053 | 1059 | 1059 | 1107 | 959 | 1014 | 1029 |
| ＞60 | 642 | 298 | 561 | 540 | 653 | 523 | 591 | 573 |

NOTE: FPKM, fragments per kilobase of transcript per million fragments mapped.
