# Supplementary figures and images for: Differentially expressed platelet activation-related genes in dogs with stage B2 myxomatous mitral valve disease
Source: BMC Vet Res. 2023 Dec 13;19:271. doi: 10.1186/s12917-023-03789-9 (PMC10717932; doi:10.1186/s12917-023-03789-9)

Module–trait relationships

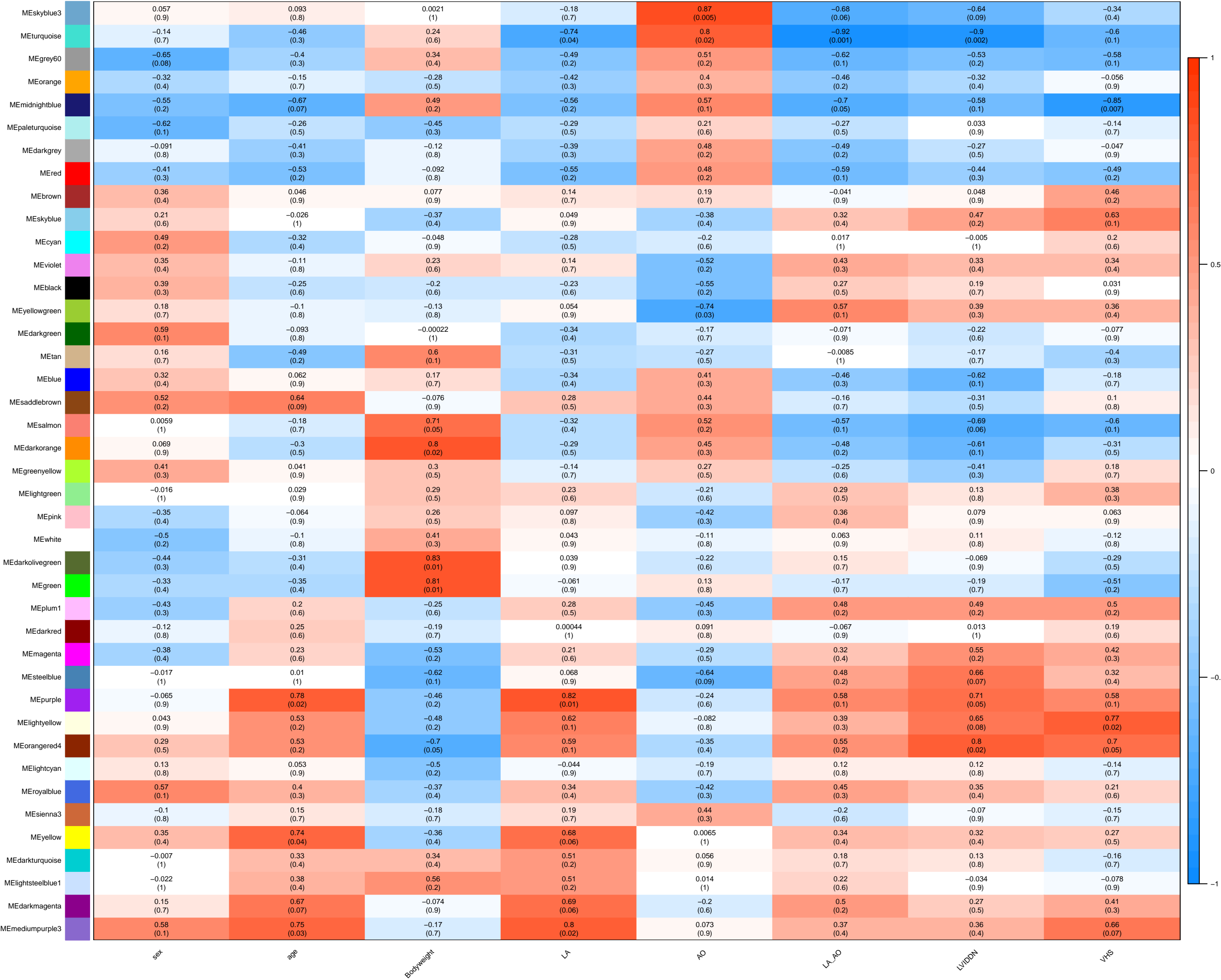

Supplement: Supplementary file 4 — Additional file 4. Module-trait relationships. [file 12917_2023_3789_MOESM4_ESM.pdf]
