# Supplementary material for: Differentially expressed platelet activation-related genes in dogs with stage B2 myxomatous mitral valve disease
Source: BMC Vet Res. 2023 Dec 13;19:271. doi: 10.1186/s12917-023-03789-9 (PMC10717932; doi:10.1186/s12917-023-03789-9)
Supplement: Supplementary file 5 — Additional file 5. The Gene information of the top 100 “hub genes” in turquoise module. [file 12917_2023_3789_MOESM5_ESM.docx]

**Additional file 5:** The Gene information of the top 100 “hub genes” in turquoise module

| Gene ID | Gene name | | K Total | | K Within | | | K Out | | | K Diff | |
| --- | --- | --- | --- | --- | --- | --- | --- | --- | --- | --- | --- | --- |
| ENSCAFG00000007885 | Q1ERZ1_CANFA | | 681.0105 | 394.2458 | | | | | 286.7647 | 107.4811 | |  |
| ENSCAFG00000005253 | | WDR43 | 660.9161 | 393.8617 | | | 267.0543 | | | 126.8074 | |  |
| ENSCAFG00000017156 | | SHC1 | 656.9403 | 388.7877 | | 268.1525 | | | | 120.6352 | |  |
| ENSCAFG00000014046 | | RASAL2 | 672.0853 | 386.8332 | | 285.2521 | | | | 101.5811 | |  |
| ENSCAFG00000015937 | | AHCTF1 | 650.055 | 382.9174 | | 267.1375 | | | | 115.7799 | |  |
| ENSCAFG00000005809 | | TM9SF2 | 660.552 | 381.8001 | | 278.7519 | | | | 103.0482 | |  |
| ENSCAFG00000024739 | | - | 652.1495 | 379.3266 | | 272.8229 | | | | 106.5037 | |  |
| ENSCAFG00000018420 | | SMCHD1 | 635.6837 | 378.2918 | | 257.3919 | | | | 120.8999 | |  |
| ENSCAFG00000024010 | | TLR1 | 660.9719 | 378.0942 | | 282.8777 | | | | 95.21654 | |  |
| ENSCAFG00000030110 | | - | 686.4352 | 376.5497 | | 309.8855 | | | | 66.66417 | |  |
| ENSCAFG00000008410 | | RASA1 | 649.7273 | 376.4974 | | 273.2299 | | | | 103.2674 | |  |
| ENSCAFG00000010292 | | DOCK10 | 688.3413 | 376.4395 | | 311.9019 | | | | 64.53764 | |  |
| ENSCAFG00000003009 | | SNX14 | 662.1948 | 371.3612 | | 290.8336 | | | | 80.52765 | |  |
| ENSCAFG00000019070 | | CHAF1A | 651.738 | 370.4259 | | 281.3121 | | | | 89.11375 | |  |
| ENSCAFG00000012884 | | RORC | 694.0901 | 369.8814 | | 324.2087 | | | | 45.67276 | |  |
| ENSCAFG00000018578 | | ZNF131 | 666.8552 | 366.1789 | | 300.6762 | | | | 65.50272 | |  |
| ENSCAFG00000012418 | | SSB | 622.8994 | 363.3635 | | 259.5359 | | | | 103.8276 | |  |
| ENSCAFG00000013761 | | CCAR1 | 708.045 | 362.8913 | | 345.1537 | | | | 17.73756 | |  |
| ENSCAFG00000020011 | | DBT | 614.9085 | 362.5157 | | 252.3928 | | | | 110.1229 | |  |
| ENSCAFG00000005054 | | ZRANB3 | 620.2309 | 361.7339 | | 258.497 | | | | 103.237 | |  |
| ENSCAFG00000005651 | | MKKS | 652.9047 | 361.2983 | | 291.6064 | | | | 69.69188 | |  |
| ENSCAFG00000011105 | | SNAP23 | 639.6535 | 358.6233 | | 281.0302 | | | | 77.59306 | |  |
| ENSCAFG00000008487 | | PDE3B | 627.6299 | 357.8471 | | 269.7828 | | | | 88.06429 | |  |
| ENSCAFG00000029142 | | C5orf51 | 640.2982 | 357.8346 | | 282.4636 | | | | 75.37094 | |  |
| ENSCAFG00000003710 | | AIM1 | 655.9039 | 357.5191 | | 298.3848 | | | | 59.13428 | |  |
| ENSCAFG00000009053 | | CKAP5 | 659.108 | 357.366 | | 301.742 | | | | 55.62402 | |  |
| ENSCAFG00000032191 | | MAP1A | 609.094 | 356.8941 | | 252.1999 | | | | 104.6942 | |  |
| ENSCAFG00000014949 | | PRRC2C | 625.1378 | 356.7868 | | 268.351 | | | | 88.43575 | |  |
| ENSCAFG00000026732 | | - | 602.0435 | 355.3478 | | 246.6957 | | | | 108.6521 | |  |
| ENSCAFG00000020250 | | - | 600.2812 | 355.0075 | | 245.2737 | | | | 109.7338 | |  |
| ENSCAFG00000029639 | | - | 606.2543 | 354.3283 | | 251.926 | | | | 102.4023 | |  |
| ENSCAFG00000018554 | | ZFP161 | 608.6271 | 353.1675 | | 255.4596 | | | | 97.70791 | |  |
| ENSCAFG00000006200 | | PLXNC1 | 646.0926 | 351.6219 | | 294.4708 | | | | 57.15111 | |  |
| ENSCAFG00000017535 | | KIF23 | 604.0733 | 350.8708 | | 253.2025 | | | | 97.66828 | |  |
| ENSCAFG00000030025 | | FGL2 | 633.7303 | 350.4044 | | 283.3259 | | | | 67.07846 | |  |
| ENSCAFG00000005702 | | TOP2B | 646.7469 | 349.1535 | | 297.5934 | | | | 51.56013 | |  |
| ENSCAFG00000028870 | | RAB6C | 632.1629 | 348.9094 | | 283.2534 | | | | 65.65602 | |  |
| ENSCAFG00000010836 | | CENPE | 599.5434 | 348.4035 | | 251.1398 | | | | 97.26371 | |  |
| ENSCAFG00000030979 | | - | 595.3698 | 348.4013 | | 246.9685 | | | | 101.4328 | |  |
| ENSCAFG00000013106 | | ATF7IP | 694.8517 | 348.3989 | | 346.4528 | | | | 1.946081 | |  |
| ENSCAFG00000023992 | | Q2Q420_CANFA | 636.4499 | 348.1187 | | 288.3311 | | | | 59.7876 | |  |
| ENSCAFG00000014175 | | ITGA4 | 596.0737 | 347.8646 | | 248.2091 | | | | 99.65554 | |  |
| ENSCAFG00000010696 | | ADD3 | 664.7073 | 346.622 | | 318.0853 | | | | 28.53675 | |  |
| ENSCAFG00000018156 | | SMG1 | 672.0732 | 346.4411 | | 325.6321 | | | | 20.809 | |  |
| ENSCAFG00000011399 | | RASSF8 | 591.4969 | 346.0978 | | 245.3991 | | | | 100.6987 | |  |
| ENSCAFG00000031918 | | TMEM26 | 591.4969 | 346.0978 | | 245.3991 | | | | 100.6987 | |  |
| ENSCAFG00000001434 | | KDM4C | 654.0405 | 344.1304 | | 309.9102 | | | | 34.2202 | |  |
| ENSCAFG00000025580 | | FRY | 623.4392 | 343.7553 | | 279.6839 | | | | 64.07132 | |  |
| ENSCAFG00000024660 | | MYOM2 | 577.1402 | 343.1613 | | 233.979 | | | | 109.1823 | |  |
| ENSCAFG00000005632 | | KAT6A | 640.4329 | 343.1291 | | 297.3039 | | | | 45.82516 | |  |
| ENSCAFG00000009321 | | RIPK1 | 618.648 | 342.104 | | 276.544 | | | | 65.56004 | |  |
| ENSCAFG00000015862 | | FAM214A | 599.9439 | 340.7299 | | 259.214 | | | | 81.51584 | |  |
| ENSCAFG00000008039 | | DIP2B | 634.356 | 338.6282 | | 295.7278 | | | | 42.90042 | |  |
| ENSCAFG00000019843 | | ANKRD11 | 691.2119 | 337.0035 | | 354.2084 | | | | -17.2049 | |  |
| ENSCAFG00000018282 | | ROCK1 | 628.3575 | 336.0015 | | 292.356 | | | | 43.64555 | |  |
| ENSCAFG00000010876 | | TET2 | 589.9919 | 335.6408 | | 254.351 | | | | 81.28979 | |  |
| ENSCAFG00000030456 | | LZIC | 635.1168 | 334.0013 | | 301.1154 | | | | 32.8859 | |  |
| ENSCAFG00000008118 | | ARRDC3 | 655.3414 | 333.7683 | | 321.5731 | | | | 12.19518 | |  |
| ENSCAFG00000020361 | | FUBP1 | 663.068 | 332.99 | | 330.078 | | | | 2.911957 | |  |
| ENSCAFG00000031079 | | ZFP36L2 | 682.6896 | 332.9023 | | 349.7873 | | | | -16.885 | |  |
| ENSCAFG00000019273 | | - | 723.4245 | 332.8049 | | 390.6196 | | | | -57.8147 | |  |
| ENSCAFG00000013226 | | PAFAH1B2 | 721.832 | 332.6741 | | 389.1579 | | | | -56.4838 | |  |
| ENSCAFG00000010807 | | PDCD4 | 677.7414 | 332.1852 | | 345.5562 | | | | -13.371 | |  |
| ENSCAFG00000004190 | | ARHGP35 | 629.8695 | 331.842 | | 298.0276 | | | | 33.81441 | |  |
| ENSCAFG00000005091 | | SNRPA | 627.8322 | 331.8188 | | 296.0134 | | | | 35.80535 | |  |
| ENSCAFG00000008788 | | MSH3 | 615.8332 | 331.8071 | | 284.0261 | | | | 47.78098 | |  |
| ENSCAFG00000028502 | | MUCSM_CANFA | 599.6903 | 331.5459 | | 268.1444 | | | | 63.40141 | |  |
| ENSCAFG00000010725 | | GIT2 | 598.1203 | 330.5263 | | 267.594 | | | | 62.93233 | |  |
| ENSCAFG00000006097 | | ASTE1 | 597.9494 | 330.46 | | 267.4894 | | | | 62.97063 | |  |
| ENSCAFG00000018963 | | ATF7IP2 | 589.3284 | 330.1842 | | 259.1442 | | | | 71.03997 | |  |
| ENSCAFG00000029403 | | - | 577.1897 | 329.5705 | | 247.6192 | | | | 81.95131 | |  |
| ENSCAFG00000006262 | | DNAJC13 | 658.9261 | 328.9174 | | 330.0087 | | | | -1.09128 | |  |
| ENSCAFG00000002998 | | ANKRD17 | 599.6915 | 328.4453 | | 271.2462 | | | | 57.19916 | |  |
| ENSCAFG00000011830 | | PITPNB | 639.2967 | 326.6577 | | 312.6389 | | | | 14.01882 | |  |
| ENSCAFG00000014016 | | DLAT | 612.2562 | 326.3393 | | 285.9169 | | | | 40.42237 | |  |
| ENSCAFG00000009323 | | UEVLD | 602.0249 | 326.2214 | | 275.8035 | | | | 50.41783 | |  |
| ENSCAFG00000018587 | | STAG2 | 636.8482 | 324.5755 | | 312.2727 | | | | 12.30279 | |  |
| ENSCAFG00000002261 | | FAM120A | 628.061 | 324.542 | | 303.519 | | | | 21.02297 | |  |
| ENSCAFG00000000418 | | MDM2 | 733.5987 | 323.9542 | | 409.6445 | | | | -85.6904 | |  |
| ENSCAFG00000007878 | | SLC43A3 | 606.4811 | 323.6892 | | 282.7918 | | | | 40.8974 | |  |
| ENSCAFG00000006456 | | RBM27 | 637.1442 | 323.048 | | 314.0962 | | | | 8.951822 | |  |
| ENSCAFG00000011407 | | SEC24B | 646.4643 | 322.2608 | | 324.2035 | | | | -1.94275 | |  |
| ENSCAFG00000008426 | | CNOT1 | 642.1073 | 321.6229 | | 320.4844 | | | | 1.138546 | |  |
| ENSCAFG00000007250 | | IFT88 | 545.9814 | 321.2789 | | 224.7025 | | | | 96.57636 | |  |
| ENSCAFG00000029211 | | RBAK | 579.6682 | 321.0091 | | 258.6592 | | | | 62.3499 | |  |
| ENSCAFG00000009236 | | CHD6 | 569.59 | 320.604 | | 248.986 | | | | 71.61793 | |  |
| ENSCAFG00000016944 | | ASH1L | 717.8995 | 320.393 | | 397.5065 | | | | -77.1135 | |  |
| ENSCAFG00000004034 | | EMP3 | 597.8561 | 320.0773 | | 277.7788 | | | | 42.29853 | |  |
| ENSCAFG00000005872 | | ARHGDIA | 616.0101 | 319.8858 | | 296.1243 | | | | 23.76146 | |  |
| ENSCAFG00000003007 | | USP34 | 589.9049 | 319.1225 | | 270.7824 | | | | 48.34017 | |  |
| ENSCAFG00000004354 | | PHTF2 | 603.6252 | 318.5136 | | 285.1115 | | | | 33.40211 | |  |
| ENSCAFG00000000079 | | KIAA1468 | 606.9824 | 315.9857 | | 290.9967 | | | | 24.98902 | |  |
| novel.18 | | - | 644.2899 | 314.8825 | | 329.4074 | | | | -14.5249 | |  |
| ENSCAFG00000015607 | | TADA1 | 613.3288 | 314.5003 | | 298.8286 | | | | 15.67171 | |  |
| ENSCAFG00000018814 | | SAFB2 | 592.6038 | 314.1363 | | 278.4675 | | | | 35.66879 | |  |
| ENSCAFG00000001459 | | CEP41 | 565.935 | 313.9287 | | 252.0063 | | | | 61.92244 | |  |
| ENSCAFG00000014975 | | EIF4G3 | 599.3713 | 313.186 | | 286.1853 | | | | 27.0007 | |  |
| ENSCAFG00000011680 | | ATP11B | 657.161 | 312.8855 | | 344.2755 | | | | -31.39 | |  |
| ENSCAFG00000017097 | | OAZ2 | 613.8239 | 312.5969 | | 301.227 | | | | 11.36992 | |  |
| ENSCAFG00000011520 | | TTC14 | 594.311 | 312.3768 | | 281.9341 | | | | 30.44269 | |  |

NOTE: k-Total, network connectivity; k-Within, intramodular connectivity; k-Out, connectivity of outside the module; k-Diff, the difference between k-within and k-out.
