# Supplementary material for: Differentially expressed platelet activation-related genes in dogs with stage B2 myxomatous mitral valve disease
Source: BMC Vet Res. 2023 Dec 13;19:271. doi: 10.1186/s12917-023-03789-9 (PMC10717932; doi:10.1186/s12917-023-03789-9)
Supplement: Supplementary file 6 — Additional file 6. The list of top 50 upregulated and downregulated DEGs. [file 12917_2023_3789_MOESM6_ESM.docx]

**Additional file 6:** The list of top 50 upregulated and downregulated DEGs

| Gene ID | log2FoldChange | *p*-value | Gene name | up/down |
| --- | --- | --- | --- | --- |
| novel.252 | 8.238538 | 0.001341 | - | up |
| ENSCAFG00000015268 | 6.413227 | 2.30E-05 | TBX19_CANFA | up |
| ENSCAFG00000029526 | 6.186295 | 0.000188 | LBX2 | up |
| ENSCAFG00000010665 | 5.694393 | 0.002055 | SLC16A14 | up |
| ENSCAFG00000015282 | 5.660419 | 0.001482 | GGT6 | up |
| ENSCAFG00000031931 | 5.497618 | 0.002233 | - | up |
| ENSCAFG00000024272 | 5.485341 | 0.030387 | - | up |
| ENSCAFG00000032691 | 5.477654 | 0.008517 | - | up |
| ENSCAFG00000029900 | 5.353028 | 0.003845 | - | up |
| ENSCAFG00000012663 | 5.348099 | 0.005152 | GPC1 | up |
| ENSCAFG00000014102 | 5.320812 | 0.005171 | ADAM11 | up |
| ENSCAFG00000029759 | 5.286795 | 0.005996 | HIST1H2AG | up |
| novel.8 | 5.273441 | 0.005343 | - | up |
| ENSCAFG00000029742 | 5.266842 | 0.016009 | SNORA26 | up |
| ENSCAFG00000001604 | 5.25505 | 0.000361 | PRICKLE4 | up |
| ENSCAFG00000032128 | 5.246583 | 0.008473 | - | up |
| ENSCAFG00000000630 | 5.246018 | 0.010696 | VWA7 | up |
| ENSCAFG00000012135 | 5.241869 | 0.017742 | CD164L2 | up |
| ENSCAFG00000006720 | 5.240925 | 0.007581 | AMOTL2 | up |
| ENSCAFG00000024087 | 5.193054 | 0.008147 | MB21D1 | up |
| ENSCAFG00000031818 | 5.172494 | 0.002264 | GGNBP1 | up |
| ENSCAFG00000012970 | 5.171751 | 0.036405 | MLXIPL | up |
| novel.501 | 5.170289 | 0.012792 | - | up |
| ENSCAFG00000028458 | 5.168885 | 0.006328 | - | up |
| ENSCAFG00000031754 | 5.124173 | 0.016314 | - | up |
| ENSCAFG00000030268 | 5.06864 | 0.010673 | - | up |
| ENSCAFG00000008391 | 5.059679 | 0.027988 | HEY1 | up |
| ENSCAFG00000020576 | 5.055196 | 0.041657 | cfa-mir-24-1 | up |
| ENSCAFG00000005243 | 5.052317 | 0.013798 | LRRC32 | up |
| ENSCAFG00000030320 | 5.032925 | 0.023971 | SHISA2 | up |
| ENSCAFG00000000378 | 5.031141 | 0.007373 | HELB | up |
| ENSCAFG00000017081 | 5.016236 | 0.003764 | ITGB1BP2 | up |
| ENSCAFG00000031503 | 4.997979 | 0.000819 | MAN1B1 | up |
| ENSCAFG00000012339 | 4.973958 | 0.020691 | ATF3 | up |
| ENSCAFG00000006676 | 4.972197 | 0.012981 | SYNE4 | up |
| ENSCAFG00000023575 | 4.95675 | 0.010459 | FAM188A | up |
| ENSCAFG00000028612 | 4.945202 | 0.033591 | - | up |
| ENSCAFG00000016485 | 4.918638 | 0.004763 | RAD51B | up |
| ENSCAFG00000028852 | 4.905556 | 0.025087 | TMEM139 | up |
| ENSCAFG00000013696 | 4.862094 | 0.004138 | ALS2CL | up |
| ENSCAFG00000018678 | 4.852014 | 0.029543 | SARM1 | up |
| ENSCAFG00000030491 | 4.850141 | 0.030894 | SNORA70 | up |
| ENSCAFG00000010546 | 4.849751 | 0.001226 | - | up |
| ENSCAFG00000002257 | 4.840982 | 0.036145 | ICA1 | up |
| ENSCAFG00000023602 | 4.838808 | 0.012997 | ECSCR_CANFA | up |
| ENSCAFG00000009670 | 4.837776 | 0.031331 | RBPJL | up |
| ENSCAFG00000017403 | 4.835294 | 0.030477 | NTN1 | up |
| ENSCAFG00000003813 | 4.824668 | 0.013851 | CCDC37 | up |
| ENSCAFG00000016790 | 4.806329 | 0.031174 | BNIP1 | up |
| ENSCAFG00000017068 | 4.80255 | 0.033734 | RNF222 | up |
| ENSCAFG00000028733 | -7.29781 | 3.60E-19 | - | down |
| ENSCAFG00000009880 | -6.11478 | 0.010556 | SNCA | down |
| ENSCAFG00000005505 | -5.733 | 9.18E-05 | PGM2L1 | down |
| ENSCAFG00000001827 | -5.71725 | 0.002109 | CROT | down |
| ENSCAFG00000014018 | -5.60512 | 0.000159 | - | down |
| ENSCAFG00000011229 | -5.53647 | 0.004988 | - | down |
| ENSCAFG00000007597 | -5.47294 | 0.003069 | ABCE1 | down |
| ENSCAFG00000000475 | -5.44529 | 0.00496 | PSORS1C2 | down |
| ENSCAFG00000019728 | -5.30657 | 0.00554 | THEG | down |
| novel.433 | -5.12921 | 0.032053 | - | down |
| novel.594 | -5.10212 | 0.01042 | - | down |
| novel.781 | -5.05959 | 0.035627 | - | down |
| novel.814 | -5.02707 | 0.001616 | - | down |
| ENSCAFG00000009372 | -5.01028 | 0.042496 | DBX2 | down |
| ENSCAFG00000014732 | -4.97366 | 0.041317 | - | down |
| ENSCAFG00000025377 | -4.77152 | 0.0057 | - | down |
| ENSCAFG00000006031 | -4.71779 | 0.001752 | LIG4 | down |
| ENSCAFG00000004115 | -4.54056 | 0.024823 | KIAA1549 | down |
| ENSCAFG00000026732 | -4.40606 | 0.042203 | - | down |
| ENSCAFG00000020288 | -4.39653 | 0.008616 | SNTB2 | down |
| ENSCAFG00000023531 | -4.34264 | 0.004187 | - | down |
| ENSCAFG00000032048 | -4.33145 | 0.00182 | - | down |
| ENSCAFG00000002065 | -4.23736 | 0.031424 | KIT_CANFA | down |
| ENSCAFG00000000234 | -4.18019 | 0.017775 | AHI1 | down |
| ENSCAFG00000016161 | -4.15765 | 0.037195 | FAM114A1 | down |
| ENSCAFG00000007728 | -4.12555 | 0.048811 | Q9GK56_CANFA | down |
| ENSCAFG00000009340 | -4.11749 | 0.005081 | SPTY2D1 | down |
| ENSCAFG00000002254 | -4.09919 | 0.003246 | REST | down |
| ENSCAFG00000008033 | -4.0871 | 0.035919 | NOC3L | down |
| ENSCAFG00000005032 | -4.05716 | 0.045702 | PIBF1 | down |
| ENSCAFG00000005054 | -3.99217 | 0.025577 | ZRANB3 | down |
| ENSCAFG00000012418 | -3.99133 | 0.00893 | SSB | down |
| ENSCAFG00000018578 | -3.97954 | 0.00205 | ZNF131 | down |
| ENSCAFG00000015464 | -3.97391 | 0.00283 | FAM64A | down |
| ENSCAFG00000031877 | -3.95858 | 0.021367 | TUG1_4 | down |
| ENSCAFG00000016200 | -3.92318 | 0.009553 | TCF12 | down |
| ENSCAFG00000012582 | -3.87702 | 0.015565 | - | down |
| ENSCAFG00000007273 | -3.79028 | 0.003599 | - | down |
| ENSCAFG00000002101 | -3.70292 | 0.002962 | - | down |
| ENSCAFG00000029302 | -3.69987 | 0.018392 | - | down |
| ENSCAFG00000009385 | -3.62023 | 0.021454 | CHST1 | down |
| ENSCAFG00000030013 | -3.61309 | 0.000639 | C1orf186 | down |
| ENSCAFG00000004866 | -3.59204 | 0.040895 | USP6NL | down |
| ENSCAFG00000017778 | -3.56331 | 0.048328 | GPRASP2 | down |
| ENSCAFG00000000581 | -3.54317 | 0.03041 | TFB1M | down |
| ENSCAFG00000009323 | -3.51525 | 0.006867 | UEVLD | down |
| ENSCAFG00000014011 | -3.35609 | 1.70E-07 | - | down |
| ENSCAFG00000018300 | -3.34944 | 0.012668 | PPP1R13B | down |
| novel.829 | -3.32161 | 0.018092 | - | down |
| ENSCAFG00000012823 | -3.29721 | 0.028676 | STRN3 | down |

**NOTE:** log2FoldChange, result performed by DESeq2 R/EdgeR R package; P value, p-value in statistical tests.
