# Supplementary material for: Differentially expressed platelet activation-related genes in dogs with stage B2 myxomatous mitral valve disease
Source: BMC Vet Res. 2023 Dec 13;19:271. doi: 10.1186/s12917-023-03789-9 (PMC10717932; doi:10.1186/s12917-023-03789-9)
Supplement: Supplementary file 7 — Additional file 7. The Gene information of platelet-related in KEGG and GO analysis. [file 12917_2023_3789_MOESM7_ESM.docx]

**Additional file 7:** The Gene information of platelet-related in KEGG and GO analysis

| **Gene ID** | **Gene-name** | ***p*-value** | **log2FoldChange** | **Up/Down** |
| --- | --- | --- | --- | --- |
| ENSCAFG00000018615 | JAK1 | 1.53E-02 | -1.47639 | Down |
| ENSCAFG00000024087 | MB21D1 | 8.15E-03 | 5.193054 | Up |
| ENSCAFG00000029302 | - | 1.84E-02 | -3.69987 | Down |
| ENSCAFG00000003549 | ROCK2 | 3.80E-02 | -2.50112 | Down |
| ENSCAFG00000009071 | Q3HTU8_CANFA | 3.31E-02 | 2.417812 | Up |
| ENSCAFG00000012508 | TP53BP1 | 1.89E-02 | -2.162 | Down |
| ENSCAFG00000015228 | VWF | 9.13E-03 | -1.98123 | Down |
| ENSCAFG00000011345 | ITPR2 | 3.01E-02 | -1.94784 | Down |
| ENSCAFG00000000418 | MDM2 | 2.23E-02 | -1.94205 | Down |
| ENSCAFG00000018282 | ROCK1 | 1.34E-02 | -1.92607 | Down |
| ENSCAFG00000019322 | GNG10 | 8.35E-03 | 1.873881 | Up |
| ENSCAFG00000009321 | RIPK1 | 1.14E-03 | -1.79607 | Down |
| ENSCAFG00000011105 | SNAP23 | 3.14E-02 | -1.69612 | Down |
| ENSCAFG00000010528 | DNM1L | 1.19E-02 | -1.65105 | Down |
| ENSCAFG00000003029 | IL8_CANFA | 5.86E-03 | 1.602834 | Up |
| ENSCAFG00000004190 | ARHGAP35 | 9.26E-03 | -1.5805 | Down |
| ENSCAFG00000020204 | - | 2.89E-03 | -1.56514 | Down |
| ENSCAFG00000007626 | PIK3R1 | 6.61E-04 | -1.54118 | Down |
| ENSCAFG00000006468 | NLRP6 | 2.86E-02 | -1.53796 | Down |
| ENSCAFG00000017563 | - | 8.43E-03 | -1.44455 | Down |
| ENSCAFG00000007537 | - | 7.46E-03 | -1.40566 | Down |
| ENSCAFG00000009086 | - | 4.66E-02 | -1.29948 | Down |
| ENSCAFG00000013418 | NFKBIA | 5.50E-03 | 1.254584 | Up |
| ENSCAFG00000000373 | TAB2 | 3.73E-02 | -1.23489 | Down |
| ENSCAFG00000031162 | Q3HTU8_CANFA | 4.04E-03 | 1.225264 | Up |
| ENSCAFG00000028460 | - | 4.54E-04 | -1.21344 | Down |
| ENSCAFG00000014612 | CXCR2 | 1.09E-02 | -1.1461 | Down |
| ENSCAFG00000025115 | - | 7.30E-03 | -1.10853 | Down |
| ENSCAFG00000023813 | - | 3.80E-03 | 1.107871 | Up |
| ENSCAFG00000011330 | NEK7 | 2.73E-02 | -1.08525 | Down |
| novel.87 | - | 3.15E-02 | 1.075757 | Up |
| ENSCAFG00000032226 | YWHAE | 2.03E-02 | -1.06166 | Down |
| ENSCAFG00000008527 | GUCY1A3 | 4.04E-02 | -1.05532 | Down |
| ENSCAFG00000000068 | BCL2 | 1.84E-02 | -1.03889 | Down |
| ENSCAFG00000000492 | - | 4.91E-02 | -1.03579 | Down |
| ENSCAFG00000014597 | ITGAV | 2.61E-02 | -1.03236 | Down |
| ENSCAFG00000004329 | APBB1IP | 1.76E-02 | -1.02368 | Down |
| ENSCAFG00000020324 | NFATC3 | 4.34E-02 | -1.02209 | Down |
| ENSCAFG00000015105 | BIRC3_CANFA | 4.69E-02 | -0.97446 | Down |
| ENSCAFG00000011376 | - | 1.13E-02 | -0.97119 | Down |
| ENSCAFG00000014145 | ITGA2B | 2.17E-03 | 0.869329 | Up |
| ENSCAFG00000006950 | SP1 | 2.74E-02 | -0.82118 | Down |
| ENSCAFG00000017669 | BTK | 2.83E-02 | -0.7935 | Down |
| ENSCAFG00000029897 | - | 2.39E-02 | 0.789941 | Up |
| ENSCAFG00000015398 | VDAC2 | 4.23E-02 | 0.780063 | Up |
| ENSCAFG00000002046 | GNG11 | 1.89E-02 | 0.766969 | Up |
| ENSCAFG00000015493 | NLRP1 | 2.65E-02 | 0.757163 | Up |
| ENSCAFG00000005895 | STIM1 | 1.02E-02 | 0.692441 | Up |
| ENSCAFG00000005704 | TMEM173 | 2.66E-02 | 0.687136 | Up |
| ENSCAFG00000019175 | TBXA2R | 9.35E-03 | -0.66948 | Down |
| ENSCAFG00000016279 | CASP9 | 4.23E-02 | 0.656108 | Up |
| ENSCAFG00000004011 | TBXAS1 | 5.95E-03 | 0.648023 | Up |
| ENSCAFG00000031157 | GP6 | 4.10E-02 | 0.618919 | Up |
| ENSCAFG00000017622 | PRKCB | 4.21E-02 | -0.56845 | Down |
| ENSCAFG00000023804 | HERC3 | 1.20E-02 | -0.945 | Down |
| ENSCAFG00000007069 | MSL2 | 1.50E-02 | -2.009 | Down |
| ENSCAFG00000017011 | - | 1.55E-02 | -0.981 | Down |
| ENSCAFG00000012582 | - | 1.56E-02 | -3.877 | Down |
| ENSCAFG00000000619 | UBR5 | 1.73E-02 | -1.253 | Down |
| ENSCAFG00000010598 | TRIP12 | 2.22E-02 | -0.857 | Down |
| ENSCAFG00000010110 | UBE3A | 2.52E-02 | -2.315 | Down |
| ENSCAFG00000012766 | UBE4A | 2.70E-02 | -1.321 | Down |
| ENSCAFG00000015058 | MUL1 | 2.80E-02 | 0.685 | Up |
| ENSCAFG00000005775 | - | 3.61E-02 | -1.430 | Down |

**NOTE:** log2FoldChange, result performed by DESeq2 R/EdgeR R package; P value, p-value in statistical tests.
