# Supplementary material for: Differentially expressed platelet activation-related genes in dogs with stage B2 myxomatous mitral valve disease
Source: BMC Vet Res. 2023 Dec 13;19:271. doi: 10.1186/s12917-023-03789-9 (PMC10717932; doi:10.1186/s12917-023-03789-9)
Supplement: Supplementary file 8 — Additional file 8. Primers used for qPCR. [file 12917_2023_3789_MOESM8_ESM.docx]

**Additional file 8:** Primers used for qPCR

| **Primers** | **Sequence (5’-3’)** |
| --- | --- |
| **MDM2** | F: GAAGCAGTAGCAGTGAGTCAACAGG |
|  | R: GCAATGAGGTGGAAGAGGAGGATTC |
| **ROCK1** | F: GGTGGTGATGGCTATTATGGACGAG |
|  | R: TGGTGCTACAGTGTCTCGGAGTG |
| **RIPK1** | F: GAGAGGAGGAAAGGAGACGAAGGG |
|  | R: ATTCACGCTTGGGATATGGCTTGG |
| **SNAP23**  **RAHGAP35**  **GAPDH** | F: ACGGAACTCAACAAGTGCTGTGG |
|  | R: AGCCTCGATCTCATTGCCCATTTC |
|  | F: GCTGGTGAGTCGCATCGTGAAG |
|  | R: GTCAATGTGGCTGGTGGCATCC |
|  | F: TTCACCACCATGGAGAAGGC |
|  | R: AGTGATGGCATGGACTGTGG |

NOTE: F, forward primer; R, Reverse primer.
